# The photosynthesis apparatus of European mistletoe (*Viscum album*)

**DOI:** 10.1093/plphys/kiac377

**Published:** 2022-08-17

**Authors:** Lucie Schröder, Jan Hegermann, Patrick Pille, Hans-Peter Braun

**Affiliations:** Institut für Pflanzgenetik, Leibniz Universität Hannover, Herrenhäuser Str. 2, 30419 Hannover, Germany; Institut für Funktionelle und Angewandte Anatomie, Medizinische Hochschule Hannover, Carl-Neuberg-Straße 1, 30625 Hannover, Germany; Institut für Pflanzgenetik, Leibniz Universität Hannover, Herrenhäuser Str. 2, 30419 Hannover, Germany; Institut für Pflanzgenetik, Leibniz Universität Hannover, Herrenhäuser Str. 2, 30419 Hannover, Germany

## Abstract

European mistletoe (*Viscum album*) is known for its special mode of cellular respiration. It lacks the mitochondrial NADH dehydrogenase complex (Complex I of the respiratory chain) and has restricted capacities to generate mitochondrial adenosine triphosphate (ATP). Here, we present an investigation of the *V. album* energy metabolism taking place in chloroplasts. Thylakoids were purified from young *V. album* leaves, and membrane-bound protein complexes were characterized by Blue native polyacrylamide gel electrophoresis as well as by the complexome profiling approach. Proteins were systematically identified by label-free quantitative shotgun proteomics. We identified >1,800 distinct proteins (accessible at https://complexomemap.de/va_leaves), including nearly 100 proteins forming part of the protein complexes involved in the light-dependent part of photosynthesis. The photosynthesis apparatus of *V. album* has distinct features: (1) comparatively low amounts of Photosystem I; (2) absence of the NDH complex (the chloroplast pendant of mitochondrial Complex I involved in cyclic electron transport (CET) around Photosystem I); (3) reduced levels of the proton gradient regulation 5 (PGR5) and proton gradient regulation 5-like 1 (PGRL1) proteins, which offer an alternative route for CET around Photosystem I; (4) comparable amounts of Photosystem II and the chloroplast ATP synthase complex to other seed plants. Our data suggest a restricted capacity for chloroplast ATP biosynthesis by the photophosphorylation process. This is in addition to the limited ATP supply by the mitochondria. We propose a view on mistletoe’s mode of life, according to which its metabolism relies to a greater extent on energy-rich compounds provided by the host trees.

## Teaser

European mistletoe not only has a very special type of cellular respiration, but also an unusual apparatus for the light-dependent part of photosynthesis.

## Introduction

European mistletoe (*Viscum album*) has been studied for more than 2,000 years (Luther and Becker, 1987). It is known for its very special life cycle (reviewed in Zuber, 2004). *Viscum album* is an obligate hemiparasitic evergreen plant that grows on branches of various trees. It is connected to the xylem of the host tree and is thus supplied with water, minerals, and to some degree with organic compounds by the host. At the same time, *V. album* carries out photosynthesis for de novo biosynthesis of organic compounds.

Photosynthesis takes place in the chloroplasts. The chloroplast ultrastructure of *V. album* resembles one of the typical seed plants (Hudák and Lux, 1986; Tuquet and Sallé, 1996; Zuber, 2004). All pigments required for photosynthesis are present (Becker, 1986) but the amounts of chlorophyll *a* as well as chlorophyll *b* are comparatively low (Hudák and Lux, 1986). The photosynthesis rate of *V. album* is comparatively low (Tuquet and Sallé, 1996; Zuber, 2004). The molecular composition of the photosynthesis apparatus of *V. album* has not been characterized so far.

In plants, photosynthesis is tightly linked to cellular respiration, which takes place in the mitochondria. Numerous metabolic pathways in leaf cells involve both the mitochondria and the chloroplasts, like photorespiration, nitrogen assimilation, heme biosynthesis, or the regulation of the redox state of the plant cell (Møller et al., 2021). The respiratory electron transfer chain is similarly composed in plants and other clades of multicellular eukaryotes: It is based on the presence of four enzyme complexes termed Complexes I, II, III, and IV; Electron transfer by Complexes I–IV is linked to the formation of a proton gradient across the inner mitochondrial membrane; the proton motive force is used by the ATP synthase complex (also designated Complex V) to generate ATP from ADP and phosphate; ATP is finally provided by the mitochondria to the entire cell and drives numerous molecular functions. However, in contrast to several other clades of multicellular eukaryotes, the mitochondria of plants comprise a series of additional so-called “alternative” respiratory enzymes, like alternative NAD(P)H dehydrogenases, or an alternative oxidase, AOX (reviewed in Schertl and Braun, 2014). These alternative enzymes take part in respiratory electron transport but do not contribute to the proton gradient across the inner mitochondrial membrane and therefore not to the formation of ATP. Their physiological roles are still under debate but seem to be relevant for keeping the redox state of the plant cell in a balance, particularly when photosynthesis takes place.

Surprisingly, it was found that cellular respiration follows unique routes in *V. album* and related species of the *Viscum* genus. Initially, it was reported that several genes are absent in the mitochondrial genomes of *Viscum* species, which code for subunits of the NADH dehydrogenase complex (Complex I) of the respiratory chain (Petersen et al., 2015a; Skippington et al., 2015; Skippington et al., 2017). It was later shown that the entire enzyme complex, which is composed of close to 50 protein subunits in plants (Klusch et al., 2021), is absent in *V. album* (MacLean et al., 2018; Senkler et al., 2018; Petersen et al., 2022; Schröder et al., 2022a). Currently, mistletoe species are the only examples of multicellular organisms that can carry out cellular respiration in the absence of Complex I (Petersen et al., 2020). To compensate for Complex I deficiency, the respiratory chain of *V. album* is elaborately rearranged: numerous alternative respiratory enzymes are present and two of the “classical” complexes of the respiratory chain, Complexes III and IV, form an especially stable supercomplex, which has been suggested to promote efficient electron transport in the terminal half of the respiratory electron transfer chain. Still, the capacity of synthesizing mitochondrial ATP is considered to be limited in *V. album*.

How can *V. album* cope with a reduced capacity for generating mitochondrial ATP? This question has been intensively discussed (Busch, 2018; da Fonseca-Pereira et al., 2018; Maclean et al., 2018; Senkler et al., 2018). One of the most ATP-consuming processes in the cytosol of *V. album* leaf cells is the synthesis of sucrose from UDP-glucose and fructose (formation of UDP glucose requires UTP, which is synthesized by UDP phosphorylation using ATP). Provision of sucrose and other sugars by the host trees, especially in spring, should lessen the ATP requirement of *V. album*. Furthermore, the growth rate of *V. album* is extremely low, which may further reduce its ATP requirement. Finally, ATP biosynthesis could be increased in other subcellular compartments in *V. album*; particularly, ATP biosynthesis by glycolysis in the cytosol or by the photophosphorylation process of the chloroplasts might be enhanced. The latter two processes have not been characterized in *V. album* on a molecular scale. Sequencing of the chloroplast genome of *V. album* revealed absence of genes encoding subunits of the chloroplast NDH complex (the chloroplast pendant of mitochondrial NADH dehydrogenase complex), which is involved in cyclic electron transport (CET) around Photosystem I (Petersen et al., 2015b). This protein complex additionally is composed of nuclear-encoded subunits, which also might be absent.

To further investigate the energy metabolism of *V. album*, we report a molecular characterization of the photosynthesis apparatus of its chloroplasts. Thylakoids were isolated from young *V. album* leaves and membrane-bound protein complexes solubilized by mild nonionic detergents. The protein complex composition of the thylakoid fraction was subsequently analyzed by 2D Blue native (BN)/SDS polyacrylamide gel electrophoresis (PAGE) and by the complexome profiling approach (van Strien et al., 2021; Wittig and Malacarne, 2021) in combination with systematic protein identifications by mass spectrometry (MS). We define a close to complete set of proteins involved in the light reaction of photosynthesis and photophosphorylation. The photosystem apparatus of *V. album* turned out to be very special. Photosystem II amounts are similar to those reported for other seed plants, but Photosystem I amounts are reduced. The ATP synthase complex of *V. album* is remarkably stable. We present evidence that *V. album* lacks the entire NDH complex, which should limit CET around Photosystem I. However, based on alternative enzymes, ATP formation by CET can still take place, but capacity for photophosphorylation should be restricted. Furthermore, redox regulation in the chloroplasts and mitochondria of *V. album* seems to be limited. These findings further demonstrate that molecular processes in *V. album* follow particular routes.

## Results

### Isolation of thylakoid membranes

**Figure 1:**
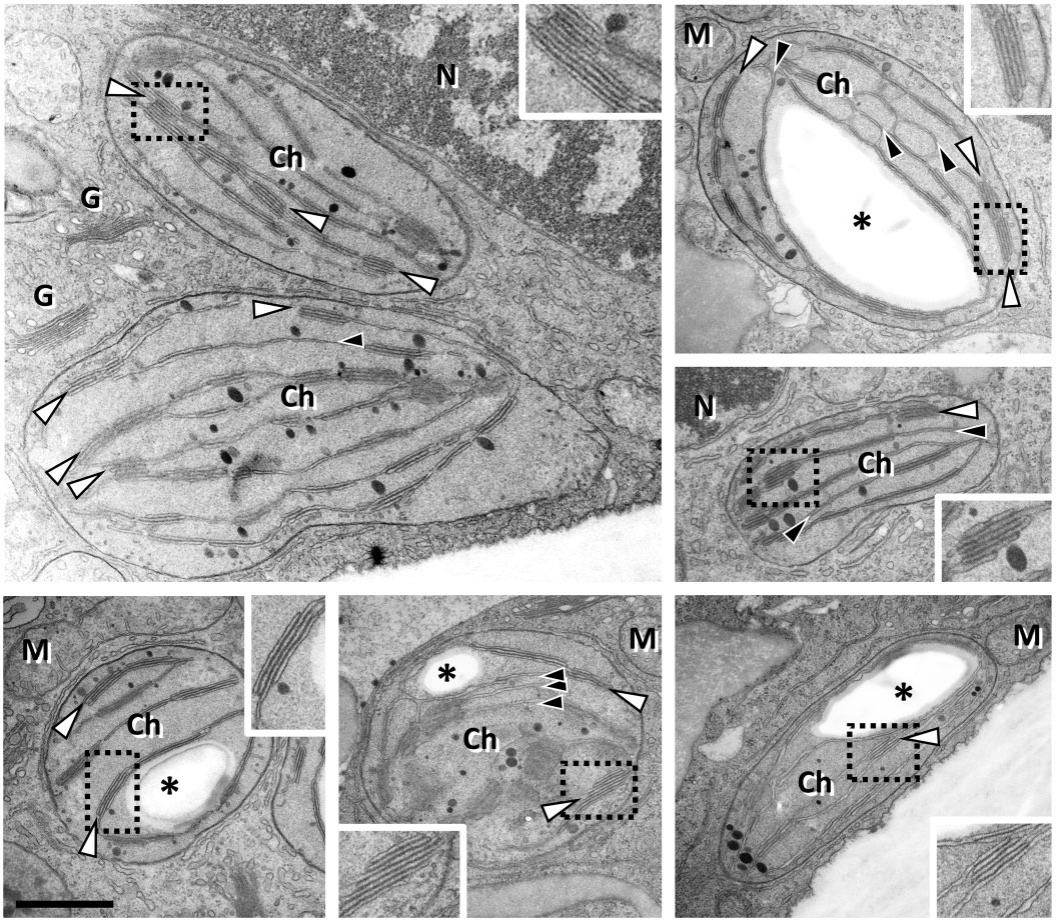
Chloroplasts in *V. album* leaf cells as revealed by transmission electron microscopy. Chloroplasts (Ch) contain single layered thylakoids (black arrowheads), lipid droplets (black dots) as well as grana (white arrowheads) consisting of stacked thylakoids. In some sections a single starch granule (asterisks) is visible. The boxed areas are shown in the insets in double magnification. N, nucleus; M, mitochondria; G, Golgi. Scale bar: 1 µm for all images; 0.5 µm for all insets.

*Viscum album* leaves were used as starting material for thylakoid preparations. For reference, thylakoids were isolated from the model plant Arabidopsis (*Arabidopsis thaliana*). For both species, actively growing leaves were chosen (see “Materials and methods” section). In *V. album*, new leaves emerge in late winter and have maximal growth rates in spring (Urech et al., 2004). The *V. album* leaves were harvested before the host tree developed leaves to avoid shading effects. Leaves include mostly oval-shaped chloroplasts as evaluated by transmission electron microscopy analysis (Figure 1). Thylakoids either are single-layered or stacked in grana with up to ten individual layers. For *A. thaliana*, rosette leaves were harvested in the middle of their growth period at 4 weeks after germination (Boyes et al., 2001). *Arabidopsis thaliana* as a reference system offers the most detailed background on the composition of the plant photosynthesis apparatus. The procedure for thylakoid isolation was identical for both species and included differential centrifugation steps and Percoll gradient centrifugation (see “Materials and methods” section). Resulting fractions were highly enriched in chloroplast proteins, especially in proteins present in the thylakoids (for purity evaluation of the fractions see below).

### Separation of thylakoid protein complexes

**Figure 2:**
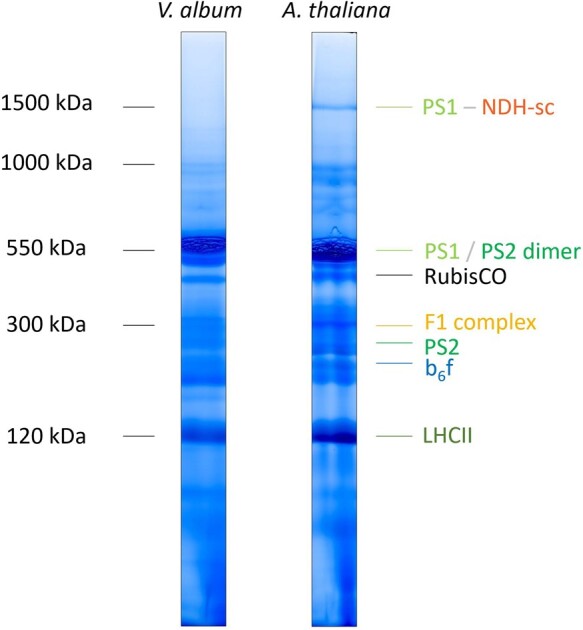
Analysis of chloroplast protein complexes from *A. thaliana* and *V. album* by 1D BN-PAGE. Thylakoid membranes were solubilized using DDM and 1D BN-PAGE was carried out as described in “Materials and methods” section. Gel lanes were Coomassie-stained. The molecular masses of standard protein complexes are given to the left of the gel lanes (in kDa) and the identity of the chloroplast protein complexes from *A. thaliana* to the right (protein complex identification is based on comparison with reference gels, Järvi et al., 2011). Designations: PS1, Photosystem I; PS2, Photosystem II; NDH, chloroplast Complex I (chloroplast NADH dehydrogenase-like complex); RubisCO, Ribulose-1,5-bisphosphate-carboxylase/-oxygenase; F_1_ complex, F_1_ part of the chloroplast ATP synthase; b_6_f, cytochrome b_6_f complex; LHCII, Light-harvesting Complex II; PSI-NDH-sc, supercomplex (sc) of NDH and two copies of monomeric PS1. The colors correspond to those given in Figures 2, 6, 8, 10, and Supplemental Figures 2 and 3.

Thylakoid fractions from *V. album* and *A. thaliana* were solubilized using dodecylmaltoside (DDM) and protein complexes were separated by BN-PAGE. For *A. thaliana*, protein complexes are visible in the 120–1,500 kDa range (Figure 2). They were identified by comparison with reference gels (Järvi et al., 2011) and by analysis using a second gel dimension (see below). For *V. album*, protein complexes are visible in the 120–1,000 kDa range.

### Subunit composition of thylakoid protein complexes in *V. album* and *A. thaliana*

**Figure 3:**
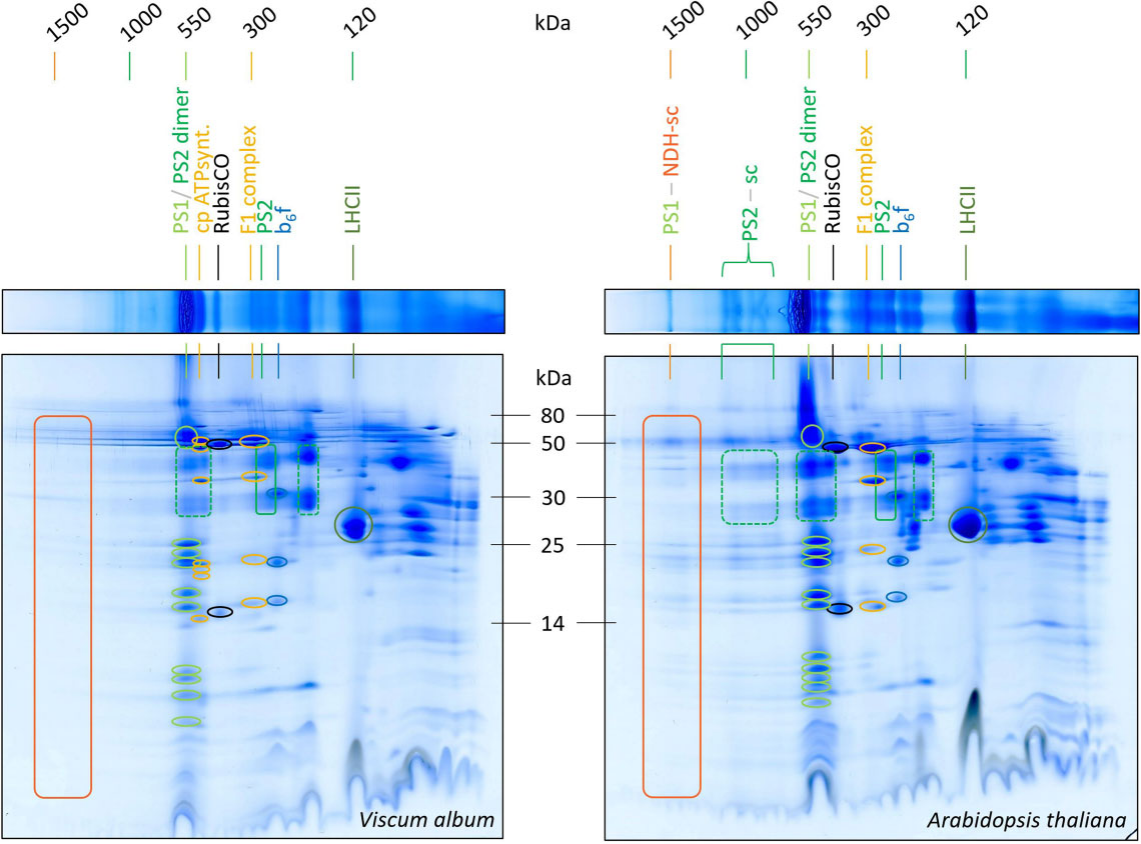
Analyses of chloroplast protein complexes from *V. album* and *A. thaliana* by 2D BN/SDS-PAGE. Lanes of 1D BN gels (Figure 2) were transferred horizontally onto SDS gels for electrophoresis in orthogonal direction (see “Materials and methods” section for details). Two-dimensional gels were Coomassie-stained. Molecular masses of standard protein complexes are given above the gels (in kDa); molecular masses of monomeric standard proteins in between the 2D gels (in kDa). The identities of protein complexes are indicated above the gels (identifications based on reference gels, see https://www.gelmap.de/arabidopsis-chloro/ and Behrens et al., 2013). For designations see legend of Figure 2. PS2-sc, supercomplexes (sc) consisting of Photosystem II. Boxes and circles on the 2D gels indicate subunits of defined protein complexes; the color code corresponds to the colors of the names of the protein complexes given above the gels. Note that the Photosystem I-NDH supercomplex and the Photosystem II supercomplexes are present in *A. thaliana* but not detectable in *V. album*.

Two-dimensional (2D) separation of thylakoid fractions from *A. thaliana* and *V. album* by BN/SDS-PAGE allowed visualizing the subunit compositions of separated protein complexes, further facilitating their identification (Figure 3). For *A. thaliana*, both photosystems, monomeric Photosystem I and the dimeric reaction center complex of Photosystem II, run at about 550 kDa. Supermolecular assemblies of Photosystem II can be detected in the 1,000 kDa range. The largest protein complex of the *A. thaliana* fraction (1,500 kDa) includes, besides Photosystem I, the chloroplast NDH complex (NADH dehydrogenase-like complex). This complex represents a homolog of the mitochondrial NADH dehydrogenase complex (Complex I of the respiratory chain); the chloroplast NDH complex therefore also is termed “chloroplast complex I.” In contrast to mitochondrial Complex I, however, the chloroplast NDH complex does not use NADH but reduced ferredoxin as an electron donor (Yamamoto et al., 2011; Schuller et al., 2019). In *A. thaliana* and other angiosperms, it has been demonstrated that the NDH complex forms a supercomplex of 1,500 kDa together with two copies of Photosystem I, which is stable upon DDM solubilization (Peng et al., 2008; Shen et al., 2022). The chloroplast NDH complex is involved in CET around Photosystem I, which contributes to the proton gradient across the thylakoid membrane and thereby to the formation of ATP (Shikanai et al., 1998; Peltier et al., 2016). In addition to monomeric Photosystem I (550 kDa), the dimeric reaction center complex of Photosystem II (550 kDa), and the Photosystem I-NDH supercomplex (1,500 kDa), the 2D BN/SDS gel of the *A. thaliana* thylakoid fraction displays the cytochrome b_6_f complex, the F_1_ part of the chloroplast ATP synthase complex, the monomeric reaction center complex of Photosystem II, the trimeric LHCII complex and some traces of RubisCO (indicating that the analyzed thylakoid fraction also includes some stromal proteins; Figure 3). Since the Photosystem I-NDH supercomplex of *A. thaliana* is of low abundance, its correct identification was verified by MS (Supplemental Figure S1).

Two-dimensional separation of thylakoid fractions from *V. album* by BN/SDS-PAGE revealed protein complexes of similar composition (Figure 3): Photosystem I and the dimeric reaction center complex of Photosystem II at 550 kDa, the cytochrome b_6_f complex, the F_1_ part of the chloroplast ATP synthase as well as the intact F_0_F_1_ ATP synthase complex, the monomeric reaction center complex of Photosystem II, the RubisCO complex and the LHCII complex. However, the Photosystem I-NDH supercomplex and the Photosystem II supercomplexes are not visible. To test if the supercomplexes might be present but of low abundance, 2D gels of the *V. album* and *A. thaliana* thylakoid fractions were repeated and silver stained (Supplemental Figure S2). Again, the Photosystem I-NAD supercomplex and the Photosystem II supercomplexes were only detectable in *A. thaliana*. Additionally, we repeated experiments using digitonin-solubilized thylakoid protein fractions. Digitonin is an especially mild detergent for thylakoid membrane solubilization (Järvi et al., 2011). In our *A. thaliana* membrane fraction, the Photosystem I-NDH supercomplex is not visible (it forms part of extremely large protein assemblies not entering the BN gel). The Photosystem II supercomplexes are nicely retained. In contrast, the Photosystem II supercomplexes and the Photosystem I-NDH supercomplex were not detectable in *V. album* (Supplemental Figure S3). We conclude that these supercomplexes are of very low abundance or absent in *V. album*.

### Analyses of thylakoid protein complexes of *V. album* and *A. thaliana* by complexome profiling

**Figure 4:**
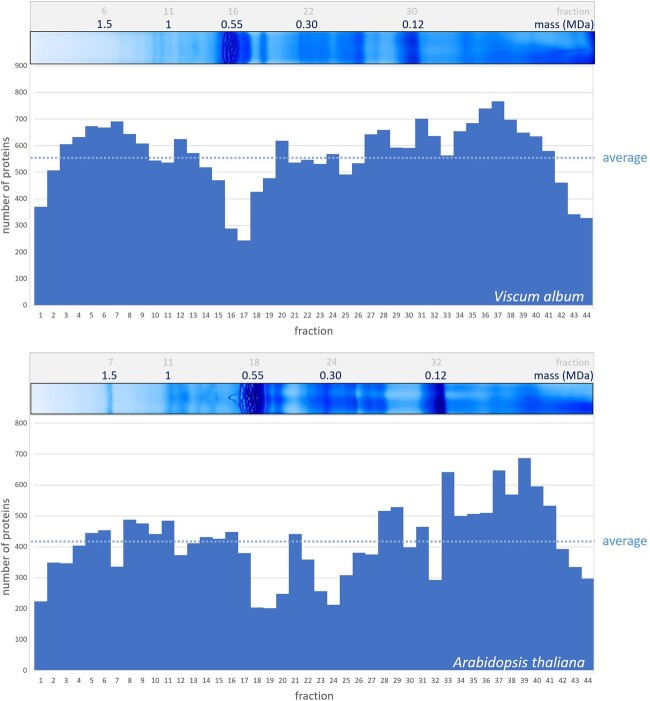
Number of proteins identified in the complexome profiling fractions of *V. album* and *A. thaliana*. BN gel lanes were each horizontally dissected into 44 gel slices, and subjected to label-free quantitative shotgun proteomics (Supplemental Figure 4). Top: Identified proteins per gel slice fraction in *V. album*; evaluation of MS data was based on the *V. album* gene space database; https://viscumalbum.pflanzenproteomik.de/, Schröder et al. (2022a). Bottom: Identified proteins per gel slice fraction in *A. thaliana*; evaluation of MS data was based on the Araport11 database (https://www.arabidopsis.org/). The lanes of the 1D BN gel used for complexome profiling are shown above the diagrams (same gel images as shown in Figure 2). MS data of *V. album* were additionally evaluated using the Araport11 database (Supplemental Figure 5).

The complexome profiling approach (van Strien et al., 2021; Wittig and Malacarne, 2021) was used to obtain deeper insights into the protein complex composition of thylakoids from *V. album*. A corresponding thylakoid fraction of *A. thaliana* was analyzed in parallel for reference. Complexome profiling allows sensitive and systematic characterization of protein complexes in cellular or subcellular fractions. It is based on protein separation by one-dimensional (1D) BN-PAGE, subsequent dissection of a BN gel lane into horizontal slices, and finally systematic protein identification in all slices by label-free quantitative shotgun proteomics. We used 44 gel slices for the thylakoid fractions from *V. album* and *A. thaliana*, respectively (Supplemental Figure S4). Data from MS were evaluated using the Araport11 database (https://www.arabidopsis.org/) for *A. thaliana* and the *V. album* gene space database (https://viscumalbum.pflanzenproteomik.de/, Schröder et al., 2022a) for *V. album*. Table 1 summarizes our results: For both species, about 400–600 proteins were detected per gel fraction (Figure 4; Supplemental Figure S5). The sum of identified proteins in the 44 fractions was 24,852 for *V. album* and 18,322 for *A. thaliana*. On average, each individual protein was identified in 13 different gel fractions (gel slices) for *V. album* and *A. thaliana*. The number of unique proteins is 1,833 for *V. album* and 1,374 for *A. thaliana*. Normalized (max) intensity profiles were calculated for all proteins along the two BN gel lanes and converted into heatmaps. In a final step, abundance profiles were aligned based on similarity using the Nova software tool (Giese et al., 2015). On the resulting figure, proteins forming part of protein complexes form clusters (Supplemental Figure S6, Supplemental Data Set S1, Supplemental Data Set S2). Evaluation of the complexome profiling data allowed defining all major protein complexes present in the thylakoids of *V. album* and *A. thaliana* (see below).

**Table 1:** Results of complexome profiling analyses for thylakoid fractions from *V. album* and *A. thaliana*

	*Viscum album*	*Arabidopsis thaliana*
Number of analyzed fractions (gel slices)	44	44
Number of identified peptides (sum of all peptides in all fractions)	136,953	99,438
Average number of peptides per fraction	3,113	2,260
Number of identified proteins (sum of all proteins in all fractions)	24,852	18,322
Average number of proteins per fraction	565	416
Number of unique peptides	12,365	10,612
Number of unique proteins	1,833	1,374
Average coverage of proteins by peptides (unique peptides / unique protein)	6.74	7.72
Average frequency of protein detection (average number of fractions, in which individual proteins were detected)	13.56	13.33

### Evaluation of the purity of the thylakoid fractions

**Figure 5:**
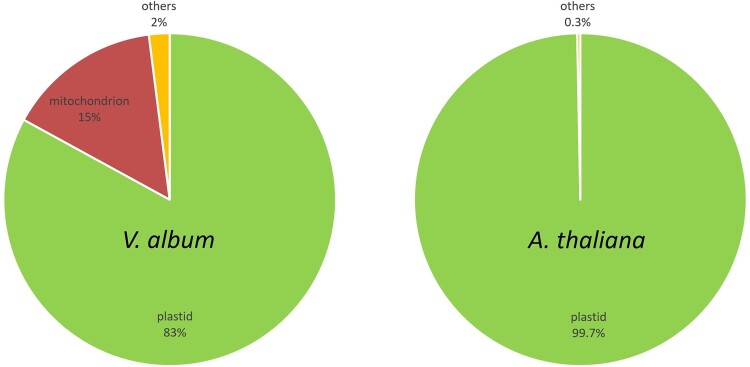
Evaluation of the purity of the thylakoid fractions from *A. thaliana* and *V. album* by cumulated protein quantities (iBAQ values) assigned to subcellular compartments according to SUBAcon (https://suba.live/). iBAQ values of all proteins identified in all complexome profiling fractions were included in this evaluation. For *V. album*, the evaluation was based on *A. thaliana* homologs of the identified proteins because SUBAcon only includes subcellular localization information for *A. thaliana*.

The MS data were also used to evaluate the purity of our thylakoid fractions. We calculated intensity-based absolute quantification (iBAQ) scores for all proteins of all complexome fractions. The iBAQ scores can be used as a quantitative estimate for each identified protein (Arike et al., 2012). In a second step, all identified proteins of all complexome profiling fractions were assigned to subcellular compartments based on the *A. thaliana* SUBAcon database (Hooper et al., 2014). This database integrates all available information on subcellular localization of all proteins for *A. thaliana*. Finally, iBAQ values of all proteins of all complexome fractions were summed up per subcellular compartment. In *A. thaliana*, >99% of the cumulated iBAQ values were assigned to the chloroplast compartment. Since the SUBAcon database only includes subcellular localization information of *A. thaliana* proteins, the same calculation could not directly be carried out for *V. album*. However, we systematically determined *A. thaliana* homologs for the identified *V. album* proteins and used these homologs for SUBAcon evaluation. >83% of the cumulated iBAQ scores of the homologs were assigned to the chloroplast compartment (Figure 5). Mitochondrial proteins represented another 15% of the cumulated iBAQ values in *V. album*. We conclude that the isolation procedure for chloroplasts, which has been optimized for *A. thaliana*, does lead to excellent results for *A. thaliana* and still very reasonable results for *V. album*. We cannot estimate the enrichment of thylakoid proteins with respect to proteins of other chloroplast subcompartments (the envelope membranes, the chloroplast stroma), since proteins of these subfractions are not defined in the SUBAcon database. However, most identified proteins clearly form part of known thylakoid protein complexes (see below).

### Subunit composition of the thylakoid protein complexes of *V. album*

**Figure 6:**
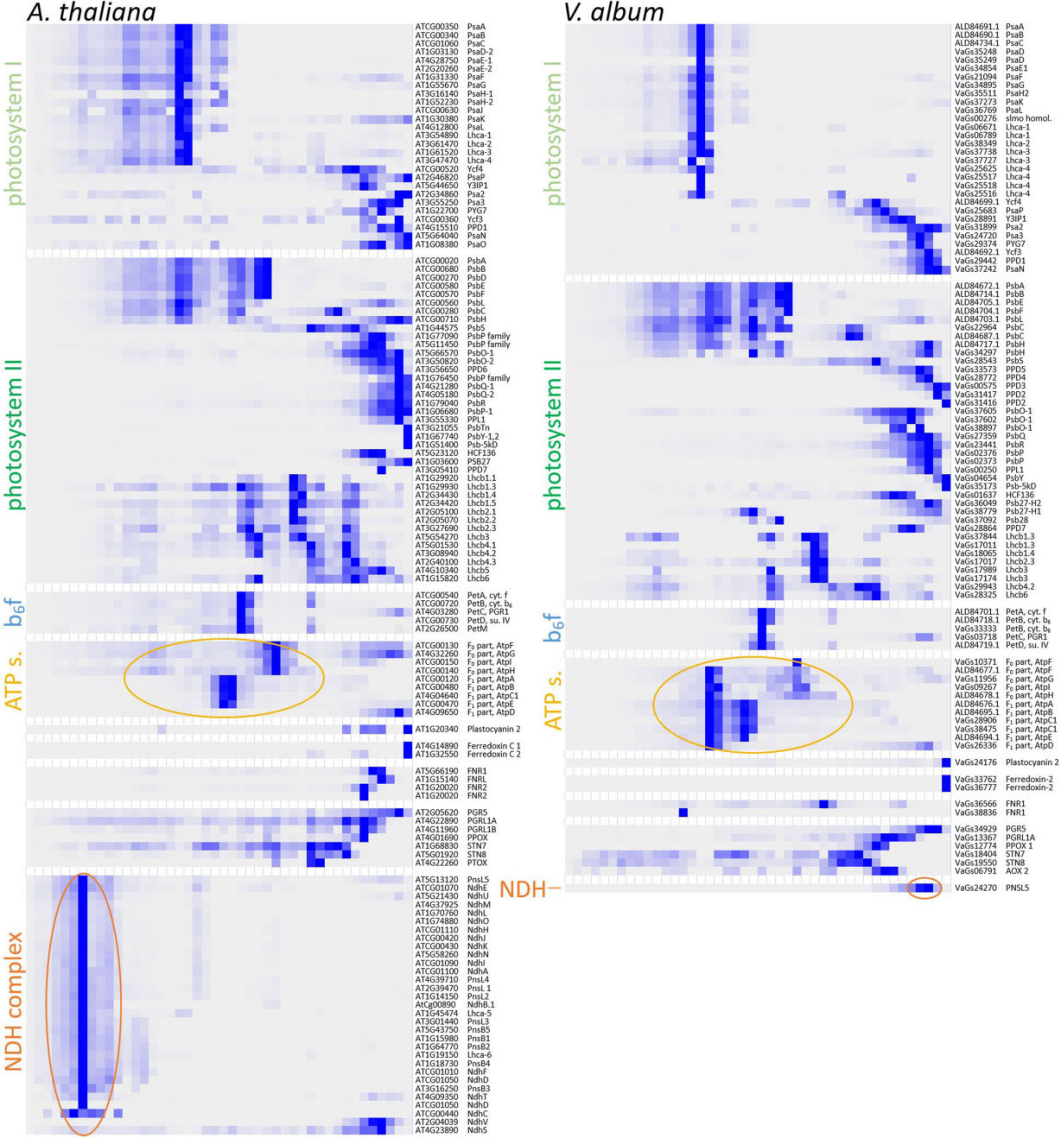
Selective display of the complexome profiling data for subunits of the Photosystem I, the Photosystem II, the cytochrome b_6_f complex, the chloroplast ATP synthase complex, the NDH complex, and some further monomeric proteins involved in photosynthesis from *A. thaliana* (left) and *V. album* (right). Relative quantities of all proteins (125 proteins in *A. thaliana* and 96 of *V. album*) along two BN gel lanes (44 fractions, respectively; Supplemental Figure 4) are displayed as heatmap (dark blue stands for high quantity, light blue/white for low quantity/no detection). For complete complexome profiling maps (1,374 proteins in *A. thaliana* and 1,833 proteins in *V. album*) see Supplemental Figure 6. Accession numbers of the proteins in the Araport11 (https://www.arabidopsis.org/) and *V. album* gene space databases (https://viscumalbum.pflanzenproteomik.de/, Schröder et al., 2022a) are given to the right of the maps; in addition, names/abbreviations of the protein names are displayed. Subunits of the chloroplast ATP synthase and the NDH complex are indicated by circles. For complete complexome profiling data see Supplemental Data Sets S1 and S2. Data also can be accessed and probed at the ComplexomeMap portal at https://complexomemap.de/va_chloroplasts and https://complexomemap.de/at_chloroplasts.

**Figure 7:**
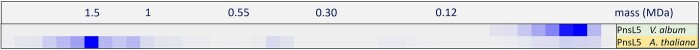
Abundance profiles of PnsL5 from *V. album* and *A. thaliana* upon complexome profiling. The molecular masses of standard protein complexes (in MDa) are given above the profiles.

The protein complexes of the thylakoids of *A. thaliana*, Photosystems I and II, the cytochrome b_6_f complex, the chloroplast ATP synthase complex, and the NDH complex, all are well defined (Shikanai, 2016; Berger et al., 2020; Malone et al., 2021). Nearly all subunits of these protein complexes were identified in the course of our complexome profiling analysis (Figure 6; Supplemental Data Set S2). Subunits of the individual protein complexes form distinct clusters on the maps. The complexome profiling map for the thylakoids of *V. album* resembles the one of the *A. thaliana* but also shows clear differences. Clusters for Photosystem I, Photosystem II, and the cytochrome b_6_f complex are similar (Figure 6; Supplemental Data Set S1, Supplemental Data Set S2). Photosystem II supercomplexes are visible in *A. thaliana* and, using the more sensitive complexome profiling approach, also in *V. album*. Compared to *A. thaliana*, the F_0_F_1_ ATP synthase complex of *V. album* is clearly more stable. Upon DDM solubilization, this complex largely is dissected into the F_0_ and F_1_ parts in *A. thaliana* (Figure 6). In *V. album*, the F_0_F_1_ holocomplex largely remains intact. This difference also is visible on the 2D BN/SDS gels for DDM-treated (Figure 3) and digitonin-treated (Supplemental Figure S3) thylakoid fractions of *V. album* and *A. thaliana*. The protein cluster of the Photosystem I-NDH supercomplex is completely absent in *V. album* (Figure 6). Subunits of the NDH complex were not detected in any *V. album* complexome fractions with the exception of the PnsL5 subunit (see below). The NDH complex of *A. thaliana* is composed of about 30 subunits (see Shikanai, 2016 and Shikanai and Aro, 2016 for review). Besides complexome profiling analyses, we systematically searched for homologs of the *A. thaliana* NDH complex in the *V. album* gene space database (https://viscumalbum.pflanzenproteomik.de/; Schröder et al., 2022a). Of the 30 subunits of the *A. thaliana* NDH complex, only one subunit, the PnsL5 protein, is present in *V. album*. This protein is an auxiliary subunit of the NDH complex (Sirpiö et al., 2009; Shikanai and Aro, 2016) and has been shown to exhibit peptidyl-prolyl isomerase activity (Edvardsson et al., 2003; Shapiguzov et al., 2006). In the *A. thaliana* complexome, PnsL5 forms part of the 1,500 kDa Photosystem I-NDH complex cluster. In contrast, in *V. album*, it clusters with monomeric proteins in the < 100 kDa range (Figure 7).

### Photosystem I has reduced abundance in *V. album*

To estimate the abundance and stoichiometry of the protein complexes involved in photosynthesis, summed up iBAQ values were calculated for selected protein complexes and related to the total iBAQ values of the analyzed thylakoid membrane fraction (Table 2). As expected, Photosystem II, which consists of the reaction center complex, the oxygen-evolving complex, and light-harvesting complexes, and which is responsible for thylakoid membrane stacking, constitutes a major part of the proteins in our thylakoid fraction (49% in *A. thaliana* and 43% in *V. album*). The cytochrome b_6_f complex represents about 3% of the total thylakoid protein in *A. thaliana* and *V. album* (it has fewer subunits and is much smaller than Photosystem II) and the ATP synthase complex constitutes about 12% of the thylakoid proteins in both species. In contrast to the three discussed complexes, the abundance of Photosystem I differs in *A. thaliana* and *V. album* (21% versus 12%). This result also is visible on the 2D BN/SDS gels (Figure 3; Supplemental Figures S2 and S3). We conclude that *V. album* has comparatively low amounts of Photosystem I and a reduced Photosystem I/Photosystem II ratio.

**Table 2:** Summed up iBAQ values of individual thylakoid protein complexes in relation to the total iBAQ value of the corresponding thylakoid membrane fraction (summed up iBAQ values of all proteins of the analyzed fraction in percent)

	*Arabidopsis thaliana*	*Viscum album*
Photosystem I	20.7	12.0
Photosystem II	49.5	43.4
Cytochrome b_6_f complex	3.2	3.0
ATP synthase	11.7	12.5

## Discussion

### Insights into the subunit composition and stoichiometry of the thylakoid protein complexes in *V. album*

Our complexome profiling analyses allowed identification of close to 100 distinct *V. album* proteins involved in the light reaction of photosynthesis. Overall, we identified >1,800 proteins in the *V. album* thylakoid fraction, which is more than the >1,300 proteins that were identified in parallel for the thylakoid fraction of the model plant *A. thaliana*. This, however, might partially reflect that the thylakoid fraction of *V. album* had a lower purity (it included some proteins of the mitochondria). Besides complexome profiling, we additionally used the amino acid sequences of the proteins involved in the light reaction of photosynthesis in *A. thaliana* to systematically probe the *V. album* gene space database (Table 3). The table includes a close to complete set of proteins involved in the light reaction in *A. thaliana* and *V. album*, most of which also were identified by complexome profiling. Several of the involved proteins occur in isoforms, especially in *V. album*.

**Table 3:** Proteins of the photosynthesis apparatus of *V. album* and *A. thaliana* detected by complexome profiling and searches in proteome databases

Protein Name (Abbreviation)	Protein Complex/Full Protein Name	Accession in *A. thaliana*	Identified in the Complexome in *A. thaliana*	Accessions in *V. album* (Accessions Identified in the *V. album* Complexome [Next Column] Are Indicated in Bold)	Identified in the Complexome in *V. album*
Photosystem I					
Lhca-1	Photosystem I	At3g54890	X	VaGs05700; VaGs05736; VaGs05756; VaGs05759; VaGs05818; VaGs05838; VaGs06232; **VaGs06671**; VaGs06787; **VaGs06789**	X
Lhca-2	Photosystem I	At3g61470	X	VaGs38348; **VaGs38349**	X
Lhca-3	Photosystem I	At1g61520	X	VaGs37725; **VaGs37727**; **VaGs37738**; VaGs37739; VaGs37740; VaGs37741	X
Lhca-4	Photosystem I	At3g47470	X	VaGs24169; VaGs24170; VaGs24171; VaGs24240; VaGs24241; VaGs24242; VaGs24243; **VaGs25516**; **VaGs25517**; **VaGs25518**; VaGs25519; VaGs25621; VaGs25623; VaGs25624; **VaGs25625**	X
Lhca-5	Photosystem I	At1g45474	X		
Lhca-6	Photosystem I	At1g19150	X		
PPD1	Photosystem I	At4g15510	X	VaGs29436; VaGs29437; VaGs29438; VaGs29439; VaGs29440; VaGs29441; **VaGs29442**	X
Psa2	Photosystem I	AT2G34860	X	VaGs31638; VaGs31897; VaGs31898; **VaGs31899**; VaGs32548	X
Psa3	Photosystem I	At3g55250	X	**VaGs24720**; VaGs25455	X
PsaA	Photosystem I	AtCg00350	X	VaGs09326; VaGs09330; VaGs09568; VaGs09860; VaGs10294; VaGs10458; VaGs10648; **ALD84691.1**	X
PsaB	Photosystem I	AtCg00340	X	VaGs09565; VaGs09569; VaGs09633; VaGs09861; VaGs10079; VaGs10295; VaGs10297; VaGs10456; VaGs10461; **ALD84690.1**	X
PsaC	Photosystem I	AtCg01060	X	**ALD84734.1**	X
PsaD-1	Photosystem I	At4g02770		**VaGs35249**; VaGs36309	X
PsaD-2	Photosystem I	At1g03130	X	VaGs35247; **VaGs35248**; VaGs35250; VaGs35312; VaGs36078; VaGs36310	X
PsaE-1	Photosystem I	At4g28750	X		
PsaE-2	Photosystem I	At2g20260	X	VaGs34853; **VaGs34854**	X
PsaF	Photosystem I	At1g31330	X	**VaGs21094**; VaGs22312; VaGs22313; VaGs22314	X
PsaG	Photosystem I	At1g55670	X	VaGs34894; **VaGs34895**; VaGs34896; VaGs34898; VaGs35931	X
PsaH-1	Photosystem I	At3g16140	X	**VaGs35511**; VaGs35660; VaGs35716; VaGs35717	X
PsaH-2	Photosystem I	At1g52230	X	VaGs35510	
PsaI	Photosystem I	AtCg00510		ALD84698.1	
PsaJ	Photosystem I	AtCg00630	X	ALD84708.1	
PsaK	Photosystem I	At1g30380	X	**VaGs37273**; VaGs37274	X
PsaL	Photosystem I	At4g12800	X	**VaGs36769**; VaGs36770; VaGs36771; VaGs37857; VaGs37858; VaGs38059; VaGs38955	X
PsaN	Photosystem I	At5g64040	X	VaGs37238; VaGs37239; **VaGs37242**	X
PsaO	Photosystem I	At1g08380	X	VaGs22666; VaGs22667; VaGs22668; VaGs23530	
PsaP	Photosystem I	At2g46820	X	**VaGs25683**; VaGs25686	X
PYG7	Photosystem I	At1g22700	X	VaGs29373; **VaGs29374**; VaGs29375	X
Y3IP1	Photosystem I	At5g44650	X	**VaGs28891**; VaGs28893; VaGs28894; VaGs30114	X
Ycf3	Photosystem I	AtCg00360	X	VaGs09567; VaGs23339; **ALD84692.1**	X
Ycf4	Photosystem I	AtCg00520	X	VaGs24236; VaGs24598; VaGs24858; VaGs24860; VaGs26085; VaGs26335; **ALD84699.1**	X
Photosystem II					
Lhcb1.1	Photosystem II	At1g29920	X		
Lhcb1.2	Photosystem II	At1g29910			
Lhcb1.3	Photosystem II	At1g29930	X	**VaGs17011**; VaGs17018; VaGs17021; VaGs18064; VaGs18184; VaGs20794; **VaGs37844**	X
Lhcb1.4	Photosystem II	At2g34430	X	**VaGs18065**; VaGs36420; VaGs36421; VaGs37845; VaGs37847; VaGs38519	X
Lhcb1.5	Photosystem II	At2g34420	X	VaGs37848	
Lhcb2.1	Photosystem II	At2g05100	X		
Lhcb2.2	Photosystem II	At2g05070	X		
Lhcb2.3	Photosystem II	At3g27690	X	VaGs17010; VaGs17016; **VaGs17017**; VaGs17020; VaGs17022; VaGs17173; VaGs17988	X
Lhcb3	Photosystem II	At5g54270	X	VaGs17015; VaGs17145; **VaGs17174**; **VaGs17989**	X
Lhcb4.1	Photosystem II	At5g01530	X	VaGs29945; VaGs30342	
Lhcb4.2	Photosystem II	At3g08940	X	VaGs29942; **VaGs29943**; VaGs29944; VaGs30341	X
Lhcb4.3	Photosystem II	At2g40100	X		
Lhcb5	Photosystem II	At4g10340	X		
Lhcb6	Photosystem II	At1g15820	X	**VaGs28325**; VaGs28326	X
PPD6	Photosystem II	At3g56650	X		
PPL1	Photosystem II	At3g55330	X	**VaGs00250**	X
PPL2	Photosystem II	At2g39470	X		
Psb-5kD	Photosystem II	At1g51400	X	**VaGs35173**	X
PsbA	Photosystem II	AtCg00020	X	**ALD84672.1**	X
PsbB	Photosystem II	AtCg00680	X	VaGs34213; VaGs34217; **ALD84714.1**	X
PsbC	Photosystem II	AtCg00280	X	VaGs22823; VaGs22824; VaGs22877; VaGs22878; VaGs22879; VaGs22956; VaGs22957; **VaGs22964**; VaGs23152; VaGs23233; VaGs23899; VaGs24088; **ALD84687.1**	X
PsbD	Photosystem II	AtCg00270	X	VaGs22880; ALD84686.1	
PsbE	Photosystem II	AtCg00580	X	VaGs24601; VaGs38380; **ALD84705.1**	X
PsbF	Photosystem II	AtCg00570	X	VaGs37226; **ALD84704.1**	X
PsbH	Photosystem II	AtCg00710	X	VaGs33255; **VaGs34297**; **ALD84717.1**	X
PsbI	Photosystem II	AtCg00080		ALD84675.1	
PsbJ	Photosystem II	AtCg00550		ALD84702.1	
PsbK	Photosystem II	AtCg00070		VaGs00341; VaGs00769; ALD84674.1	
PsbL	Photosystem II	AtCg00560	X	**ALD84703.1**	X
PsbM	Photosystem II	AtCg00220		VaGs09191; ALD84685.1	
PsbN	Photosystem II	AtCg00700		ALD84716.1	
PsbO-1	Photosystem II	At5g66570	X	VaGs36809; VaGs36810; **VaGs37602**; VaGs37604; **VaGs37605**; **VaGs38897**	X
PsbO-2	Photosystem II	At3g50820	X		
PsbP family	Photosystem II	At1g76450	X	**VaGs00575**; VaGs00577	X
PsbP family	Photosystem II	At1g69680			
PsbP family	Photosystem II	At1g77090	X	**VaGs28772**	X
PsbP family	Photosystem II	At5g11450	X	**VaGs33573**	X
PsbP family	Photosystem II	At3g05410	X	**VaGs28864**	X
PsbP-1	Photosystem II	At1g06680	X	VaGs02374; VaGs02375; **VaGs02376**	X
PsbP-2	Photosystem II	At2g30790		**VaGs02373**; VaGs03329; VaGs03330	X
PsbQ-1	Photosystem II	At4g21280	X	VaGs27356; VaGs27357; VaGs27358; **VaGs27359**; VaGs27654; VaGs28561	X
PsbQ-2	Photosystem II	At4g05180	X		
PsbR	Photosystem II	At1g79040	X	VaGs22618; VaGs23098; VaGs23099; VaGs23265; VaGs23267; VaGs23438; **VaGs23441**	X
PsbS	Photosystem II	At1g44575	X	VaGs27026; VaGs27027; VaGs27028; VaGs27030; VaGs27031; VaGs27173; VaGs28072; VaGs28170; VaGs28171; VaGs28349; **VaGs28543**; VaGs28544; VaGs28545	X
PsbT	Photosystem II	AtCg00690		ALD84715.1	
PsbTn	Photosystem II	At3g21055	X	VaGs36194; VaGs36532	
PsbW	Photosystem II	At2g30570		VaGs31144; VaGs32081; VaGs32110; VaGs32305; VaGs32438; VaGs32481	
PsbX	Photosystem II	At2g06520		VaGs38401; VaGs38404	
PsbY-1,2	Photosystem II	At1g67740	X	VaGs04653; **VaGs04654**; VaGs05309; VaGs05482; VaGs05483; VaGs05484; VaGs34835	X
PsbZ	Photosystem II	AtCg00300		VaGs23703; ALD84688.1	
cyt b6f complex					
PetA, cyt. f	cyt b6f complex	ATCG00540	X	VaGs37147; VaGs37223; **ALD84701.1**	X
PetB, cyt. b_6_	cyt b6f complex	ATCG00720	X	**VaGs33333**; VaGs34157; VaGs34706; **ALD84718.1**	X
PetC, PGR1	cyt b6f complex	AT4G03280	X	VaGs03572; VaGs03573; VaGs03574; **VaGs03718**	X
PetD, su. IV	cyt b6f complex	ATCG00730	X	VaGs33222; VaGs33251; VaGs33339; VaGs34489; **ALD84719.1**	X
PetG	cyt b6f complex	ATCG00600		VaGs24596; VaGs25230; VaGs25801; VaGs37142; ALD84707.1	
PetL	cyt b6f complex	ATCG00590		ALD84706.1	
PetM	cyt b6f complex	AT2G26500	X	VaGs38214; VaGs38215	
PetN	cyt b6f complex	ATCG00210		VaGs02857; ALD84684.1	
ND complex					
NdhA	ND complex	ATCG01100	X		
NdhB.1	ND complex	AtCg00890	X		
NdhB.2	ND complex	ATCG01250			
NdhC	ND complex	ATCG00440	X		
NdhD	ND complex	ATCG01050	X		
NdhE	ND complex	ATCG01070	X		
NdhF	ND complex	ATCG01010	X		
NdhG	ND complex	ATCG01080			
NdhH	ND complex	ATCG01110	X		
NdhI	ND complex	ATCG01090	X		
NdhJ	ND complex	ATCG00420	X		
NdhK	ND complex	ATCG00430	X		
NdhL	ND complex	AT1G70760	X		
NdhM	ND complex	AT4G37925	X		
NdhN	ND complex	AT5G58260	X		
NdhO	ND complex	AT1G74880	X		
NdhS	ND complex	AT4G23890	X		
NdhT	ND complex	AT4G09350	X		
NdhU	ND complex	AT5G21430	X		
NdhV	ND complex	AT2G04039	X		
PnsB1	ND complex	AT1G15980	X		
PnsB2	ND complex	AT1G64770	X		
PnsB3	ND complex	AT3G16250	X		
PnsB4	ND complex	AT1G18730	X		
PnsB5	ND complex	AT5G43750	X		
PnsL1	ND complex	AT2G39470	X		
PnsL2	ND complex	AT1G14150	X		
PnsL3	ND complex	AT3G01440	X		
PnsL4	ND complex	AT4G39710	X		
PnsL5	ND complex	AT5G13120	X	**VaGs24270**; VaGs25386; VaGs25641; VaGs25642; VaGs25643	X
atp-synthase					
F_0_ part, AtpI	atp-synthase	ATCG00150	X	**VaGs09267**; VaGs10676; ALD84679.1	X
F_0_ part, AtpF	atp-synthase	ATCG00130	X	VaGs09265; VaGs09681; **VaGs10371**; VaGs10673; **ALD84677.1**	X
F_0_ part AtpG	atp-synthase	AT4G32260	X	VaGs10779; **VaGs11956**; VaGs12030; VaGs12031; VaGs35192; VaGs35193	X
F_0_ part, AtpH	atp-synthase	ATCG00140	X	VaGs09832; VaGs10218; **ALD84678.1**	X
F_1_ part, AtpA	atp-synthase	ATCG00120	X	VaGs09205; VaGs09268; VaGs09285; VaGs09379; VaGs09387; VaGs09593; VaGs09762; VaGs09807; VaGs09808; VaGs09828; VaGs09979; VaGs10066; **ALD84676.1**	X
F_1_ part AtpB	atp-synthase	ATCG00480	X	VaGs27798; VaGs27799; AAK72868.1; **ALD84695.1**	X
F_1_ part AtpD	atp-synthase	AT4G09650	X	VaGs24411; VaGs24412; VaGs25113; VaGs25114; **VaGs26336**; VaGs26369	X
F_1_ part AtpE	atp-synthase	ATCG00470	X	**ALD84694.1**	X
F_1_ part AtpC1	atp-synthase	AT4G04640	X	**VaGs28906**; **VaGs38475**; VaGs38476	X
F_1_ part AtpC2	atp-synthase	AT1G15700			
plastocyanin 1	plastocyanin	AT1G76100		VaGs24175; VaGs25830	
plastocyanin 2	plastocyanin	AT1G20340	X	**VaGs24176**; VaGs26370	X
ferredoxin					
ferredoxin C 1	ferredoxin	AT4G14890	X	VaGs36776; **VaGs36777**	X
ferredoxin C 2	ferredoxin	AT1G32550	X	VaGs24838; VaGs37132	
ferredoxin 1	ferredoxin	AT1G10960		**VaGs33762**	X
ferredoxin 2	ferredoxin	At1g60950		VaGs33666; VaGs34701; VaGs35670; VaGs36681	
ferredoxin 3	ferredoxin	AT2G27510		VaGs36222	
ferredoxin 4	ferredoxin	AT5G10000			
ferredoxin-NADP+ OR					
FNR1	ferredoxin-NADP+ OR	AT5G66190	X	VaGs38832; VaGs38833	
FNR2	ferredoxin-NADP+ OR	AT1G20020	X	**VaGs36566**; **VaGs38836**; VaGs38841	X
FNRL	ferredoxin-NADP+ OR	AT1G15140	X		
others					
AOX1a	others	At3g22370		VaGs06230; VaGs06620; VaGs06621; VaGs06681; **VaGs06791**; UER43485.1	X
AOX2	others	At5g64210			
PGR5	others	AT2G05620	X	**VaGs34929**; VaGs36763	X
PGRL1A	others	AT4G22890	X	**VaGs13367**; VaGs13368; VaGs14105; VaGs14106; VaGs14107; VaGs14108; VaGs14110	X
PGRL1B	others	AT4G11960	X		
PPOX	others	AT4G01690	X	**VaGs12774**; VaGs13625	X
PTOX	others	AT4G22260	X		
STN7	others	AT1G68830	X	**VaGs18404**	X
STN8	others	AT5G01920	X	**VaGs19550**; VaGs19552	X

*Notes:* List of proteins forming part of the two photosystems, the cytochrome b_6_f complex, the chloroplast ATP synthase complex, the NDH complex, and further proteins involved in thylakoid electron transfer processes and their regulation. The known proteins from *A. thaliana* (Shikanai, 2016; Berger et al., 2020; Malone et al., 2021) were used to probe the *V. album* gene space database (https://viscumalbum.pflanzenproteomik.de/; Schröder et al., 2022a). Accessions are either from Araport (https://www.arabidopsis.org/), The *V. album* gene space database (https://viscumalbum.pflanzenproteomik.de/) or NCBI (https://www.ncbi.nlm.nih.gov/).

Photosystems I and II, together with the cytochrome b_6_f complex, are involved in linear electron transport (LET) from H_2_O to NADP^+^. As a result, NADPH and ATP are produced. Additionally, Photosystem I and the cytochrome b_6_f complex are involved in CET, which only generates ATP. The ratio of LET and CET can be adjusted by the numeric ratio of the two photosystems, but also by modulation of the antenna sizes associated with the photosystems (regulated, besides others, by the STN7 and STN8 protein kinases; see Longoni and Goldschmidt-Clermont, 2021 for review) and by regulation of photosystem activities. Interestingly, the amount of Photosystem I is comparatively low in *V. album*. Shading effects, which result in relative enrichment of far-red light and which are known to also cause a reduction of Photosystem I (reviewed in Schöttler and Tóth, 2014), can be excluded since *V. album* was harvested in spring before leaves of the host tree developed. Since Photosystem II amounts are similar in *A. thaliana* and *V. album* (Table 2), the Photosystem I–Photosystem II ratio is reduced in *V. album*, which should affect both LET and CET. This is in line with previous reports that *V. album* has a reduced Photosystem I to Photosystem II activity (Tuquet and Sallé, 1996). As a result, the overall photosynthesis rate in *V. album* is reduced (Tuquet and Sallé, 1996; Zuber, 2004). This also should affect photophosphorylation.

Photophosphorylation, the formation of ATP by phosphorylation of ADP using the proton gradient across the thylakoid membrane build up by LET and CET, is catalyzed by the chloroplast ATP synthase complex. In the chloroplasts of *A. thaliana* and *V. album*, amounts of chloroplast ATP synthase complexes are similar. This might reflect the importance of the chloroplast ATP synthase complex in regulating the pH in the thylakoid lumen (Trinh and Masuda, 2022). The chloroplast ATP synthase complex has been found to be especially stable in *V. album* (Figures 3 and 6). However, decreased LET and CET should reduce the proton gradient across the thylakoid membrane and thereby diminish the capacity to produce ATP. The proton gradient may be further reduced by the activity of the plastid terminal oxidase (PTOX), which catalyzes plastoquinol oxidation using oxygen as a direct electron acceptor. Interestingly, by our complexome profiling analysis, PTOX was identified in *A. thaliana* but not in *V. album*.

### Absence of the NDH complex and the Photosystem I-NDH supercomplex

**Figure 8:**
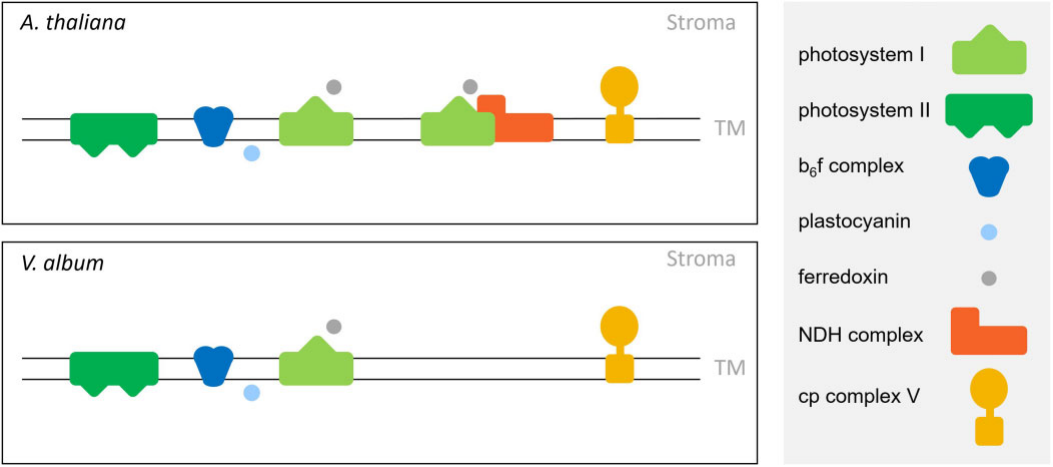
Chloroplast protein complexes involved in photosynthesis in *A. thaliana* and *V. album*. Yellow, chloroplast (cp) complex V; orange, cp Complex I (NDH complex); dark blue, cytochrome b_6_f complex; light green, Photosystem I; dark green, Photosystem II; light blue, plastocyanin; grey, ferredoxin. TM, thylakoid membrane.

The chloroplast NDH complex is absent in *V. album*. This protein complex, which is homologous to mitochondrial Complex I, includes about 30 subunits. It catalyzes ferredoxin-plastoquinone oxidoreduction and is involved in CET around the Photosystem I. Most of the subunits of this protein complex are encoded by the nuclear genome in plants, but some are encoded in the plastidic genome (reviewed in Shikanai, 2016). It has been reported previously that the plastidic genes encoding subunits of the NDH complex are absent in *Viscum* species (Petersen et al., 2015b). We here report that the entire NDH complex including the nuclear-encoded subunits is absent in *V. album* (Figure 8). Only one NDH subunit is retained in *V. album*, the PnsL5 protein. This protein is considered to be bifunctional since it exhibits peptidyl-prolyl isomerase activity and at the same time is necessary for the assembly of the NDH complex (Sirpiö et al., 2009; Shikanai and Aro, 2016). Similarly, bifunctional subunits of mitochondrial Complex I were recently reported to be retained in *V. album* despite the loss of this respiratory protein complex (Petersen et al., 2022; Schröder et al., 2022a). In the *A. thaliana* thylakoid complexome, PnsL5 forms part of the 1,500 kDa Photosystem I-NDH complex cluster. In contrast, in *V. album*, it clusters with monomeric proteins in the <100 kDa range (Figure 7). Since the NDH complex is absent in *V. album*, Photosystem I only is present as a monomer. Absence of the NDH complex should affect chloroplast capacity to re-oxidize ferredoxin and thereby CET.

### CET around Photosystem I may be mediated by the PGR5/PGRL1 proteins in *V. album*

**Figure 9:**
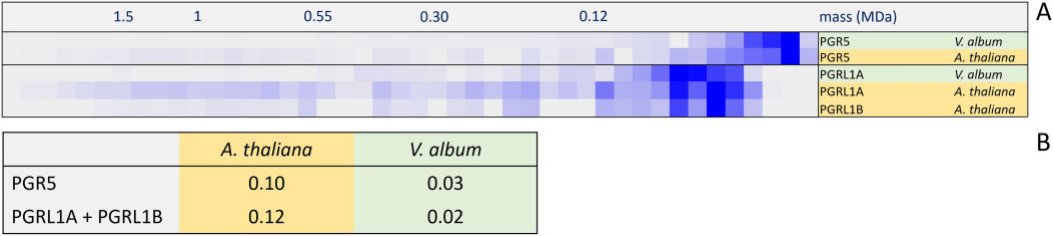
Complexome profiling results for PGR5- and PGR5-like proteins (PGRL1) of *V. album* and *A. thaliana*. A, Relative abundances of the proteins along the 1D BN gel lane used for complexome profiling. The molecular masses of standard protein complexes (in MDa) are given above the abundance profiles. B, Summed up iBAQ values of the PGR5 and PGRL1 proteins in relation to the total iBAQ values of the corresponding thylakoid fraction (percent of total protein).

**Figure 10:**
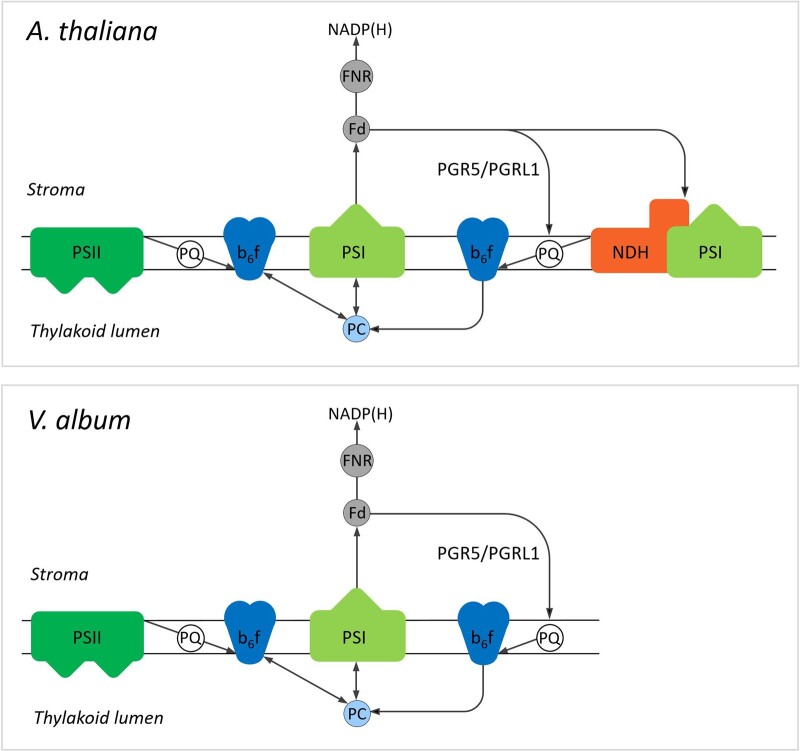
Model of the linear and CET pathways in *V. album* and *A. thaliana*. In LET, electrons originate from Photosystem II (PSII). They are transferred via plastoquinol (PQ) to the cytochrome b_6_f complex (b_6_f) and via plastocyanin (PC) from the cytochrome b_6_f complex to Photosystem I (PSI). LET terminates by electron transfer from PSI to ferredoxin (Fd) and from Fd to NADP^+^, which is reduced to NADPH (the latter step is catalyzed by ferredoxin-NADP^+^ reductase (FNR)). In contrast, in CET, electrons originate from Photosystem I. They are transferred onto Fd, but afterward not further transferred to NADP^+^, but to the cytochrome b_6_f complex. In *A. thaliana*, this electron transfer requires either the PGR5/PGRL1 proteins or the NDH complex, which forms a supercomplex with the Photosystem I. CET is completed by transfer of electrons from the cytochrome b_6_f complex via PC back to PSI. In *V. album*, electron transport from Fd to b_6_f depends entirely on PGR5/PGRL1, since the NDH complex is absent. The colors of the involved components correspond to those introduced in Figure 2. Note that further CET pathways were suggested to occur but so far could not be precisely defined (Nawrocki et al., 2019). The figure is based on Figure 1 in Johnson (2011).

In plants, CET also can be carried out by an alternative pathway involving the PGR5/PGRL1 proteins (Munekage et al., 2002; DalCorso et al., 2008; Hertle et al., 2013; reviewed in Shikanai, 2014; Ma et al., 2021). These proteins are likewise present in *A. thaliana* and *V. album*, but, compared to *A. thaliana*, amounts of these proteins are reduced in *V. album* (Figure 9). We conclude that CET can take place in *V. album* (Figure 10). However, reduced amounts of Photosystem I together with the absence of one of the two CET pathways around Photosystem I both indicate that the capacity for chloroplast ATP formation based on photophosphorylation might be restricted in *V. album*. The degree of restriction is difficult to predict because further CET pathways were suggested to occur, which so far could not be precisely defined (Nawrocki et al., 2019). Also, the molecular functions of the PGR5/PGRL1 proteins might go beyond CET (Kanazawa et al., 2017; Shimakawa and Miyake, 2018) and have to be further defined.

### Life without mitochondrial and chloroplast Complex I

*Viscum album* and some other related species of the *Viscum* and *Phoradendron* genera of the *Santalaceae* family are the only multicellular species that can carry out cellular respiration without mitochondrial Complex I (Petersen et al., 2015a, Skippington et al., 2015; Maclean et al., 2018; Senkler et al., 2018; Zervas et al., 2019; Petersen et al., 2022; Schröder et al., 2022a). In addition, *V. album* now has been shown to completely lack chloroplast Complex I, the NDH complex. Absence of NDH is less an exception than absence of mitochondrial Complex I. Indeed, loss of chloroplast Complex I has been reported for a few clades of plants (Wakasugi et al., 1994; Wicke et al., 2011; Lin et al., 2017; Silva et al., 2018; reviewed in Sabater, 2021). In general, the NDH complex is found in mosses, ferns, and seed plants, but there are exceptions that concern some aquatic plants, plant parasites, and a few further plant species that grow under comparatively mild conditions. In contrast, most algae lack the NDH complex. It has been concluded that the NDH complex is beneficial in stressful terrestrial environments to maintain efficient photosynthesis (Sabater, 2021). However, its biosynthesis requires substantial amounts of energy for protein synthesis and for assembly of the protein complex. Under mild conditions, it might be advantageous to save this energy.

The *Viscum* species are another group of organisms that do not require the NDH complex. Most remarkably, they are the only known clade of plants that simultaneously lack both the mitochondrial and the chloroplast Complex I. The absence of mitochondrial Complex I reduces the mitochondrial capacity to produce ATP. Reduced amounts of Photosystem I together with the absence of chloroplast Complex I simultaneously limits the capacity for chloroplast ATP formation. *V. album* might tolerate low ATP formation because its growth rate is low. Furthermore, the host trees provide energy-rich organic compounds, including glucose, fructose, and sucrose, especially in spring (Escher et al., 2004). Sucrose biosynthesis in the cytosol requires substantial amounts of mitochondrial ATP, which can be saved if the sucrose is provided by the host. Indeed, maximal growth of *V. album* takes place in spring, when deciduous trees supply organic compounds to their developing organs of the new growing season (Urech et al., 2004). The importance of the host trees supplying *V. album* with carbohydrates may have been underestimated so far.

### The *V. album* way of life

*Viscum album* and related species are known for their very special life cycle (Luther and Becker, 1987; Zuber, 2004). Already von Tubeuf (1922) concluded on *V. album* “Nothing about this plant is normal.” Several regulatory mechanisms seem to be diminished in *V. album*: It has no polar axis but grows in all directions resulting in the spherical shape of the adult plant; stomata are at both sides of the leaves and not (much) regulated; seed dormancy does not take place; and senescence processes are very reduced as green leaves are discarded. Meanwhile increasing information is available on the molecular biology of *V. album* at the levels of its gene space, transcriptome, proteome, and metabolome (Novák et al., 2020; Jäger et al., 2021; Schröder et al., 2022a). Mechanisms to limit an increase in genome size were obviously weak during evolution, leading to one of the largest plant genomes ever described (Zonneveld, 2010). Mitochondria lack Complex I, have decreased amounts of the mitochondrial ATP synthase complex and fewer cristae. The amounts of Photosystem I are reduced and chloroplast Complex I is absent, restricting CET at Photosystem I. Besides limited organellar ATP generation, the absence of chloroplast and mitochondrial Complex I also should affect the capacity of *V. album* to regulate its cellular redox balance. Reduced levels of regulation in general means reduced energy costs, which, however, comes with the price of reduced molecular coordination and safeguard. Overall, the *V. album* way of life seems to be characterized by a sophisticated system of controlled deregulation.

## Materials and methods

### Plant material

European Mistletoe (*V. album*) grown on an apple (*Malus domestica*) tree on our university campus (Leibniz Universität Hannover, Herrenhäuserstr. 2 in Hannover/Germany) was harvested in spring 2019. During the period of harvesting, the apple tree started to bloom but still was without leaves. The thylakoid preparation for the complexome profiling experiment was performed on April 25, 2019. Leaves were harvested at 9 am. Local weather data for Herrenhäuser Str. 2/Hannover in April 2019 are provided by the Institute of Meteorology of Leibniz Universität Hannover at https://www1.muk.uni-hannover.de/hp-design2020/wetter_archiv_frame.html. Leaves for electron microscopy analyses were harvested at 9 am. Arabidopsis (*A. thaliana*; ecotype Columbia 0) was cultivated in parallel in a phyto chamber under long-day conditions (16-h light/8-h dark; 22°C). PAR intensity was 110 µmol s^−1^ m^−2^; light source: Philips F25T8/TL841 lamps. Plants were harvested 4 weeks after germination. Leaves were used for experimental analyses.

### Transmission electron microscopy

Transmission electron microscopy of *V. album* leaf cells was carried out as described previously (Senkler et al., 2018). In brief: Freshly harvested *V. album* leaves were cut into 1 mm pieces. The pieces were fixed in 150 mM HEPES, pH 7.35, containing 1.5% [v/v] formaldehyde and 1.5% glutaraldehyde [v/v] and washed with water. Afterward, the samples were incubated for 2 h in 1% [w/v] OsO_4_ solution containing 1.5% [w/v] hexacyanoferrat II, subsequently washed with water and stored in 1% [w/v] aqueous uranyl acetate solution overnight. On the next day, the samples were washed again with water, dehydrated in acetone, and finally embedded in Low Viscosity Resin (Agar Scientific, Essex, UK). Ultrathin sections (60 nm) were mounted on formvar-coated copper grids and poststained with uranyl acetate and lead citrate (Reynolds, 1963). Samples were examined using a Morgagni Transmission Electron Microscope (FEI).

### Isolation of thylakoid membranes

*Viscum album* and *A. thaliana* leaves (30 g each) were used as starting material for the preparation of thylakoid membranes. All the following steps were carried out at 4°C. Homogenization of leaves was performed in 400 mL chilled homogenization buffer (50 mM HEPES, 2 mM EDTA, 1 mM MgCl, 5 mM sodium ascorbate, 330 mM sorbitol, 0.5% [w/v] BSA, pH 8.0 [KOH]) using a Waring blender (one pulse of 3 s at high speed, two pulses of 3 s at low speed; breaks of 30 s in between the pulses). Resulting homogenates were filtered through 2 layers of Miracloth. Filtrates were centrifuged at 300 g at 4°C for 5 min. Supernatants were removed and the pellets carefully resuspended in 2 mL of homogenization buffer using a brush. Subsequently, 10 mL chilled Percoll medium (50 mM HEPES, 330 mM sorbitol, 35% [v/v] Percoll, pH 8.0 [KOH]) was transferred into tubes for gradient centrifugation. Two to three milliliter of the resuspended samples were carefully loaded on top of the Percoll medium, respectively. Centrifugation took place at 90.000 g for 20 min at 4°C. The upper (thylakoid) band was transferred to new tubes with sorbitol-HEPES (SH) buffer (50 mM HEPES, 330 mM sorbitol, pH 8.0 [KOH]). Washing steps (at least 4) were performed in SH buffer at 1.000 g for 5 min. Supernatants were discarded after the centrifugations, respectively, and pellets were carefully dissolved in SH buffer and collected. After the last centrifugation step, pellets were dissolved in 2 mL SH buffer and divided into aliquots of 200 µL, which were either directly used for biochemical analyses (see below) or shock frozen in liquid nitrogen and stored at −80°C.

### Gel electrophoresis procedures

Isolated thylakoid fractions (200 µL) were centrifuged and pellets resuspended in 90 µL solubilization buffer DDM (25 mM BisTris/HCl pH 7.0, 20% [v/v] glycerol, 3% [w/v] dodecyl maltoside [DDM]). For digitonin solubilization, the pellets were resuspended in solubilization buffer digitonin (30 mM HEPES/HCl, pH 7.4, 150 mM potassium acetate, 10% [v/v] glycerol, 5% [w/v] digitonin). Further sample preparation and performance of 1D BN-PAGE and 2D BN/SDS-PAGE was carried out as described previously (Wittig et al., 2006). Gels were stained using the Coomassie colloidal staining procedure (Neuhoff et al., 1988). To increase staining sensitivity, selected gels were silver-stained using a modified version of a protocol published previously (Heukeshoven and Dernick, 1988). In short, gels were fixed in fixing solution (50% [v/v] ethanol, 10% [v/v] acetic acid) for 2 h. Gels were next treated with incubation solution (30% [v/v] ethanol, 0.2% [w/v] sodiumthiosulfate, 0.8 M NaAc) for 2 h and subsequently washed 3 times with ddH_2_O. Binding of silver to proteins was achieved by incubating gels with silver nitrate solution (0.1% [w/v] AgNO_3_) for 30 min. Gels were rinsed briefly with ddH_2_O and afterward washed thoroughly with a washing solution (2.5% [w/v] Na_2_CO_3_) for 1 min. Protein visualization took place in a fresh box using developing solution (2.5% [w/v] Na_2_CO_3_, 0.01% [v/v] formaldehyde). The time of development may differ and depends on the amount of protein separated within a gel (on average 10–30 min). As soon as the desired staining result has been achieved, the development process is stopped by transferring gels into stopping solution (0.05 EDTA).

### Protein identifications after gel electrophoresis

Proteins of interest were cut from the 2D gels and identified by MS as described previously (Senkler et al., 2018). For *A. thaliana*, MS data were evaluated using the *A. thaliana* Araport11 protein database (https://www.arabidopsis.org/).

### Complexome profiling

Complexome profiling is based on the separation of a complex protein sample under native conditions and the subsequent systematic analysis of several gel fractions along the native separation matrix by label-free quantitative shotgun proteomics (Arnold and Braun, 2022). We performed 1D BN-PAGE for protein separation and used 44 fractions for proteome analyses, respectively. The procedure has been described previously (Schröder et al., 2022b). We used an Ultimate 3000 UPLC/Q Exactive Orbitrap mass spectrometer (Thermo Fisher Scientific, Dreieich, Germany) for label-free quantitative shotgun proteomics. For MS data evaluation the Araport11 protein database (https://www.arabidopsis.org/) was used for *A. thaliana* and the *V. album* gene space (VaGs) database at https://viscumalbum.pflanzenproteomik.de/, Schröder et al., 2022a) for *V. album*. The following parameters were used for MS data analyses: Digestions mode: Specific; Enzyme: Trypsin/P; Maximum missed cleavage sites: 2; Variable modifications: Oxidation (M) and Acetyl (Protein N-term); Maximum number of modifications per peptide: 5. Global parameters were set to: minimal peptide length: 7; maximum peptide mass: 4,600 Da; fixed modification: Carbamidomethyl (C). The peptide-to-spectrum match and the false discovery rates were set to 1% for protein identification. The default value of 1 was used for minimum number of peptides, razor peptides, and unique peptides (0) of the protein group identification. iBAQ values (Schwanhäusser et al., 2011) were determined for all proteins in all fractions and used for the calculation of abundance profiles of proteins along the 1D BN gel dimension.

### Generation of ComplexomeMaps

Normalized (max) intensity profiles for all proteins along the two BN gel lanes were converted into heatmaps (Schröder et al., 2022b). In a final step, abundance profiles were aligned based on hierarchical clustering using the Nova software tool (Giese et al., 2015). Complexome profiling data were displayed in the form of ComplexomeMaps (https://complexomemap.de/) as described previously (Senkler et al., 2018). The *V. album* thylakoid ComplexomeMap is accessible at https://complexomemap.de/va_chloroplasts and the *A. thaliana* thylakoid ComplexomeMaps at https://complexomemap.de/at_chloroplasts.

## Accession numbers

Sequence data of this article can be found in the *V. album* gene space (VaGs) database (Schröder et al., 2022a) at https://viscumalbum.pflanzenproteomik.de/.

## Data availability Statement

Primary data of the complexome profiling experiments can be accessed at the ComplexomeMap portal at https://complexomemap.de/va_chloroplasts (*V. album*) and https://complexomemap.de/at_chloroplasts (*A. thaliana*).

Primary data of the complexome profiling experiments also can be accessed in Supplemental data Sets S1 and S2.

The mass spectrometry proteomics data have been deposited to the ProteomeXchange Consortium via the PRIDE (Perez-Riverol et al., 2022) partner repository with the dataset identifier PXD035825 and PXD035871.

## Supplemental data

The following materials are available in the online version of this article.

**Supplemental Figure S1.** Analyses of the Photosystem I-NDH supercomplex from *A. thaliana*.

**Supplemental Figure S2.** Two-dimensional analysis of thylakoid fractions from *V. album* and *A. thaliana* by BN/SDS-PAGE in combination with silver staining.

**Supplemental Figure S3.** Two-dimensional analysis of digitonin-treated thylakoid fractions from *V. album* and *A. thaliana* by 2D BN/SDS-PAGE.

**Supplemental Figure S4.** BN gel lanes of separated thylakoid protein complexes from *V. album* and *A. thaliana* used for complexome profiling.

**Supplemental Figure S5.** Number of proteins identified in the complexome profiling fractions of *V. album* from the database used for data evaluation.

**Supplemental Figure S6.** Heatmap of normalized (max) abundance profiles of thylakoid proteins from *V. album* leaves.

**Supplemental Data Set S1.** Heatmap of intensity profiles of the proteins included in the complexome dataset for thylakoids of *V. album.*

**Supplemental Data Set S2.** Heatmap of intensity profiles of the proteins included in the complexome dataset for thylakoids of *A. thaliana.*

## Supplementary Material

kiac377_Supplementary_Data

## Notes

H.-P.B. initiated and supervised the project. Isolation of chloroplasts and gel electrophoresis procedures were carried out by L.S., electron microscopy analyses by J.H., mass spectrometry analysis by P.P. All authors were involved in data evaluation and interpretation. H.P.B. and L.S. wrote the manuscript.

The author responsible for distribution of materials integral to the findings presented in this article in accordance with the policy described in the Instructions for Authors (https://academic.oup.com/plphys/pages/general-instructions) is Hans-Peter Braun: (braun@genetik.uni-hannover.de).
