# Supplementary material for: The photosynthesis apparatus of European mistletoe (Viscum album)
Source: Plant Physiol. 2022 Aug 17;190(3):1896–914. doi: 10.1093/plphys/kiac377 (PMC9614478; doi:10.1093/plphys/kiac377)
Supplement: kiac377_Supplementary_Data [file kiac377_supplementary_data.zip › Schroeder et al. 2022_Supp. Figures.pdf]

Supplemental Material

Supp. Fig. S1

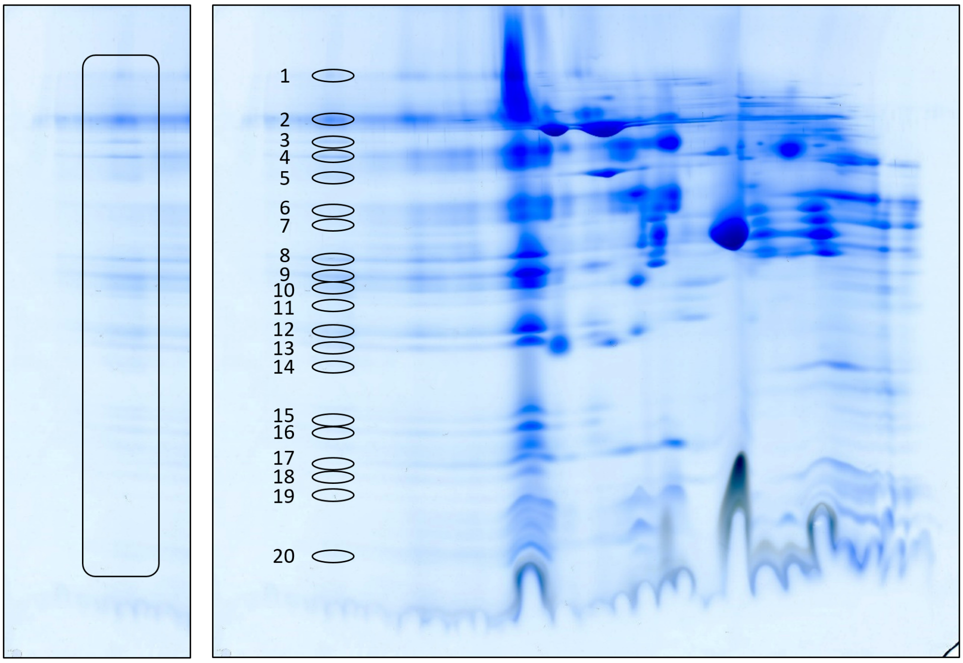

ID	Accession	Name	MW [kDa]	Mascot Score
1		not identified		
2	ATCG00350.1	PSAA	83.2	**248**
2	ATCG00340.1	PSAB	82.4	**45**
2	ATCG01010.1	NDHF	85.2	**41**
3	AT1G15980.1	PNSB1	51.0	**146**
4	ATCG01110.1	NDHH	45.5	**173**
5		not identified		
6	ATCG00270.1	PSBD	39.5	**51**
7		not identified		
8	AT1G61520.1	LHCA3	29.2	**86**
8	AT1G19150.1	LHCA6	29.9	**50**
8	AT3G01810.1	EEIG1/EHBP1 protein amino-terminal domain protein	101.6	**35**
9	AT1G45474.1	LHCA5	27.8	**113**
9	AT1G03130.1	PSAD-2	22.3	**105**
10	AT1G03130.1	PSAD-2	22.3	**162**
10	AT2G39470.1	PNSL1	26.9	**46**
11	AT2G39470.1	PNSL1	26.9	**153**
12	AT1G31330.1	PSAF	24.2	**236**
12	AT4G12800.1	PSAL	23.0	**134**
12	AT3G01440.1	PNSL3	24.8	**87**
13		not identified		
14	AT1G14150.1	PNSL2	22.1	**114**
15	AT1G52230.1	PSAH2	15.3	**66**
15	AT4G11400.1	ARID/BRIGHT DNA-binding domain	65.6	**31**
16	AT1G55670.1	PSAG	17.1	**157**
16	ATCG01070.1	NDHE	11.3	**43**
17		not identified		
18	AT1G30380.1	PSAK	13.2	**40**
19		not identified		
20		not identified		

Supp. Figure S1: Analyses of the photosystem I-NDH supercomplex from *A. thaliana*. Thylakoid membranes were solubilized using DDM and protein complexes resolved by 2D Blue native PAGE (the gel image is the same as shown in Fig. 3) The subunits of the PS I-NDH supercomplex were analyzed by mass spectrometry. Green: subunits of photosystem I; orange: subunits of the NDH complex.

Supp. Fig. S2

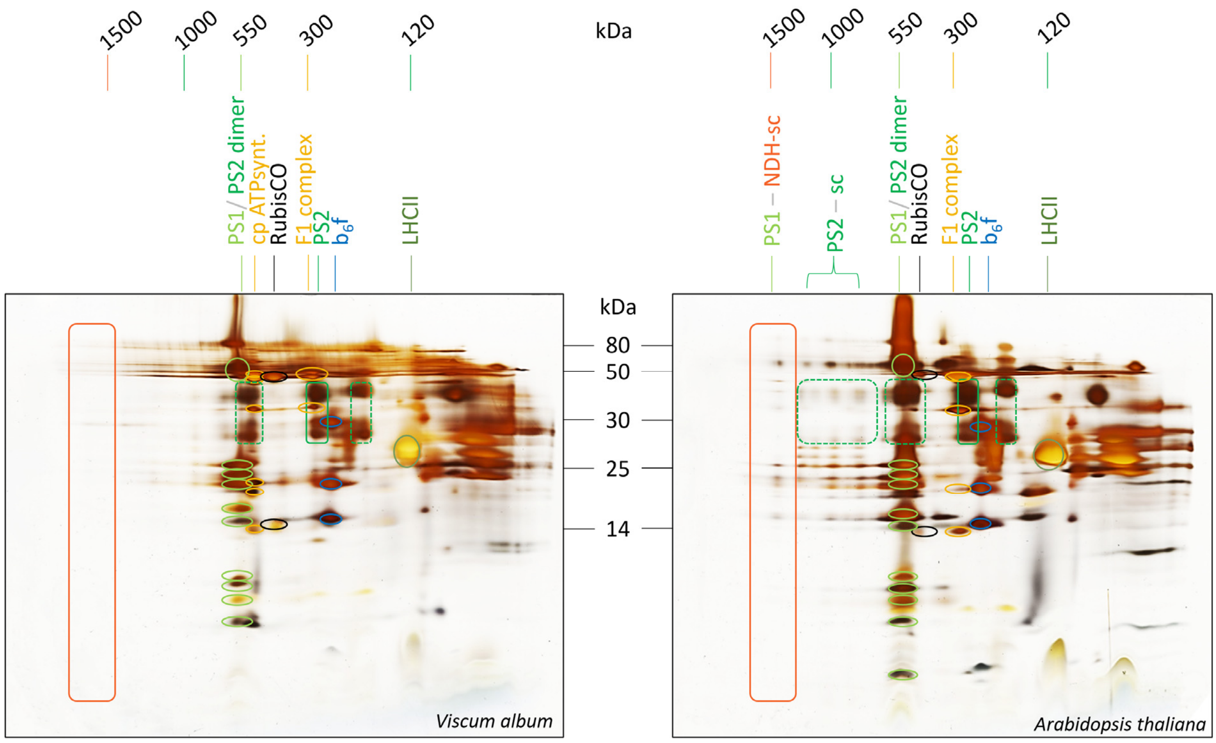

Supp. Figure S2: Two-dimensional analysis of thylakoid fractions from *V. album* and *A. thaliana* by Blue native / SDS PAGE in combination with silver staining. Thylakoid membranes were solubilized using DDM. Molecular masses of standard protein complexes are given above the gels (in kDa); molecular masses of monomeric standard proteins in between the 2D gels (in kDa). The identities of protein complexes are indicated above the gels (identifications based on reference gels; Järvi et al. 2011). Designations: PS1: photosystem I; PS2: photosystem II; NDH: chloroplast complex I (chloroplast NADH dehydrogenase-like complex); cp ATP Synthase: chloroplast ATP synthase; RubisCO: Ribulose-1,5-bisphosphat-carboxylase/-oxygenase; F_1 complex: F_1 part of the cp ATP synthase; b_6f: cytochrome b_6f complex; LHCII: light harvesting complex II; sc: supercomplex. PS1-NDH-sc: supercomplex of NDH and two copies of monomeric PS1. PS2-sc: photosystem II supercomplexes.

Supp. Fig. S3

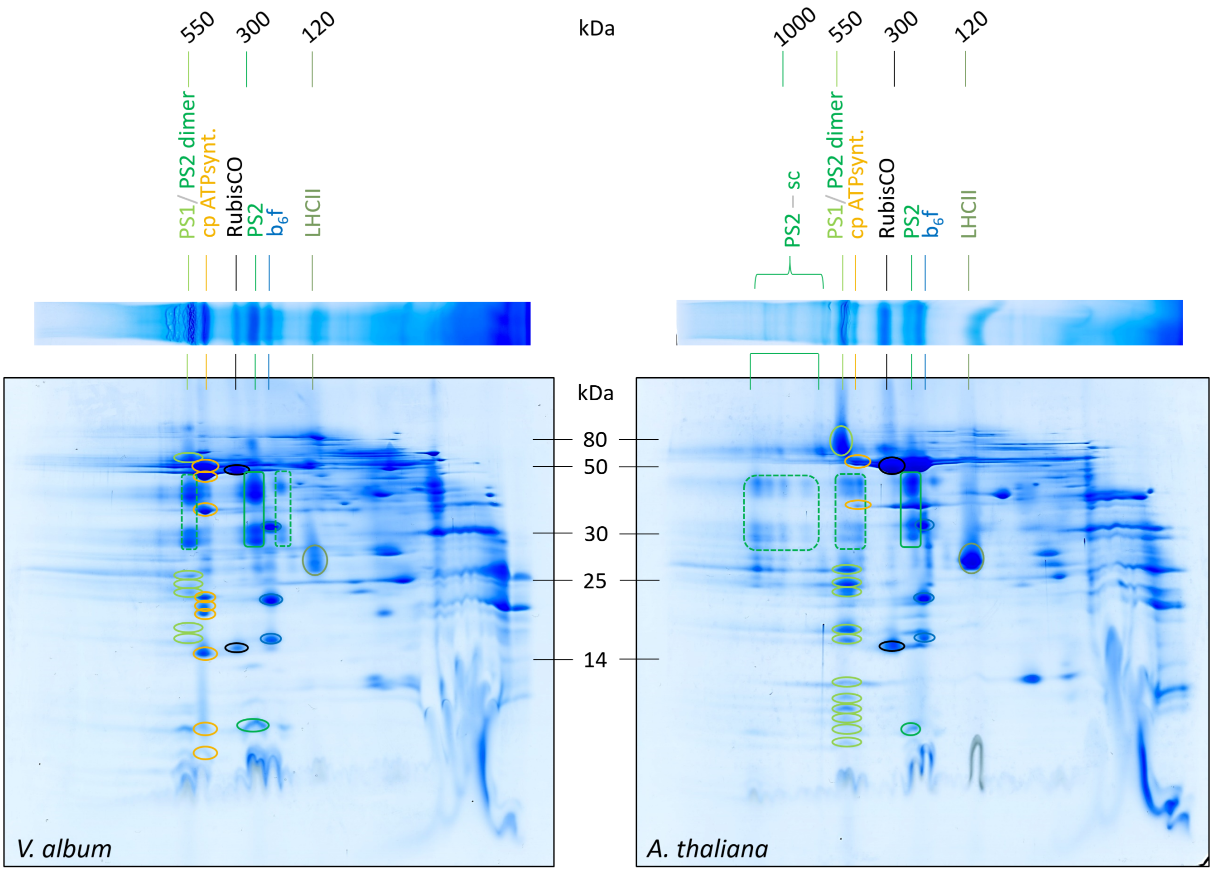

Supp. Figure S3: Two-dimensional analysis of digitonin-treated thylakoid fractions from *V. album* and *A. thaliana* by 2D Blue native / SDS PAGE. Thylakoid membranes were solubilized using 5% digitonin. The gels were Coomassie-stained. Molecular masses of standard protein complexes are given above the gels (in kDa); molecular masses of monomeric standard proteins in between two 2D gels (in kDa). The identities of protein complexes are indicated above the gels (identifications based on reference gels; Järvi et al. 2011). Designations: PS1: photosystem I; PS2: photosystem II; cp ATP Synthase: chloroplast ATP synthase; RubisCO: Ribulose-1,5-bisphosphat-carboxylase/-oxygenase; F_1 complex: F_1 part of the cp ATP synthase; b_6f: cytochrome b_6f complex; LHCII: light harvesting complex II; sc: supercomplex; PS2-sc: photosystem II supercomplexes.

Supp. Fig. S4:

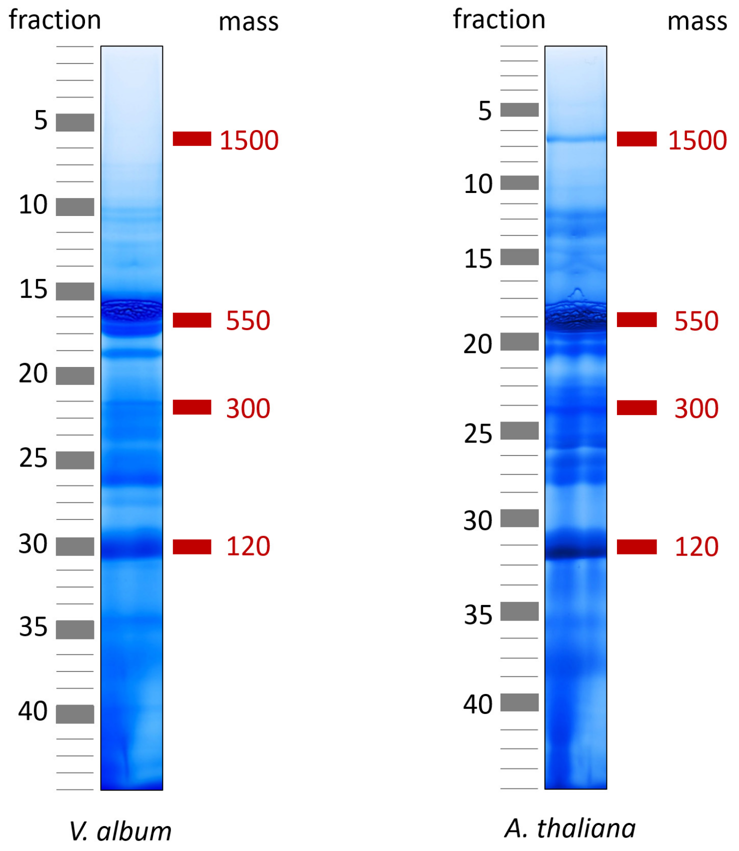

Supp. Figure S4: BN gel lanes of separated thylakoid protein complexes from *V. album* and *A. thaliana* used for complexome profiling. The molecular masses of standard protein complexes are given to the right of the gel lanes (in kDa). Both lanes were dissected into 44 gel slices, respectively, which are indicated to the left of the gel lanes. Finally, all 2 x 44 gel slices were subjected to analyses by label free quantitative mass spectrometry for systematic protein identifications. The gel images shown are the same as in Fig. 2.

Supp. Fig. S5

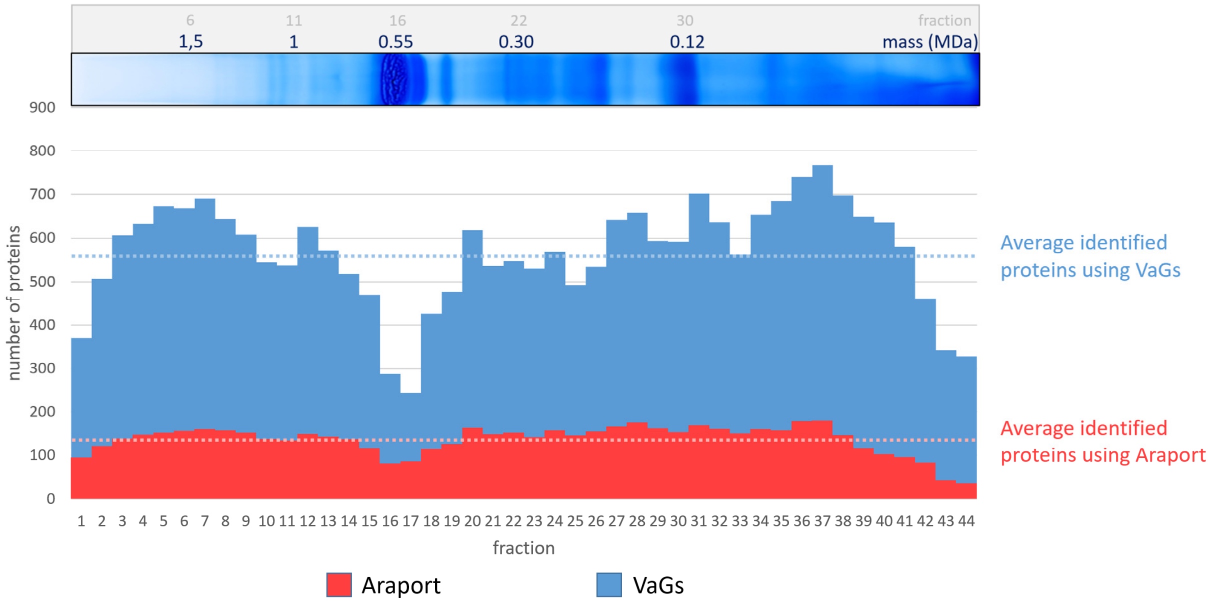

Supp. Figure S5: Number of proteins identified in the complexome profiling fractions of *V. album* in dependence of the database used for data evaluation. The BN gel lane is shown above the diagram (same gel image as the one shown in Fig. 2 for *V. album*). The lane was dissected in 44 gel slices (= fractions), which all were subjected to label free quantitative mass spectrometry (MS). Finally, MS data were evaluated using (i) the *A. thaliana* Araport11 gene database (https://www.arabidopsis.org/) (red columns) or (ii) the *V. album* protein space database (https://viscumalbum.pflanzenproteomik.de/, Schröder et al. 2022a) (blue columns). Note that the average number of identfied proteins is only 134 based on Araport11 evaluation. Evaluation using the novel *V. album* gene space database on average revealed 565 (Table 1) proteins per fraction.

Supp. Fig. S6

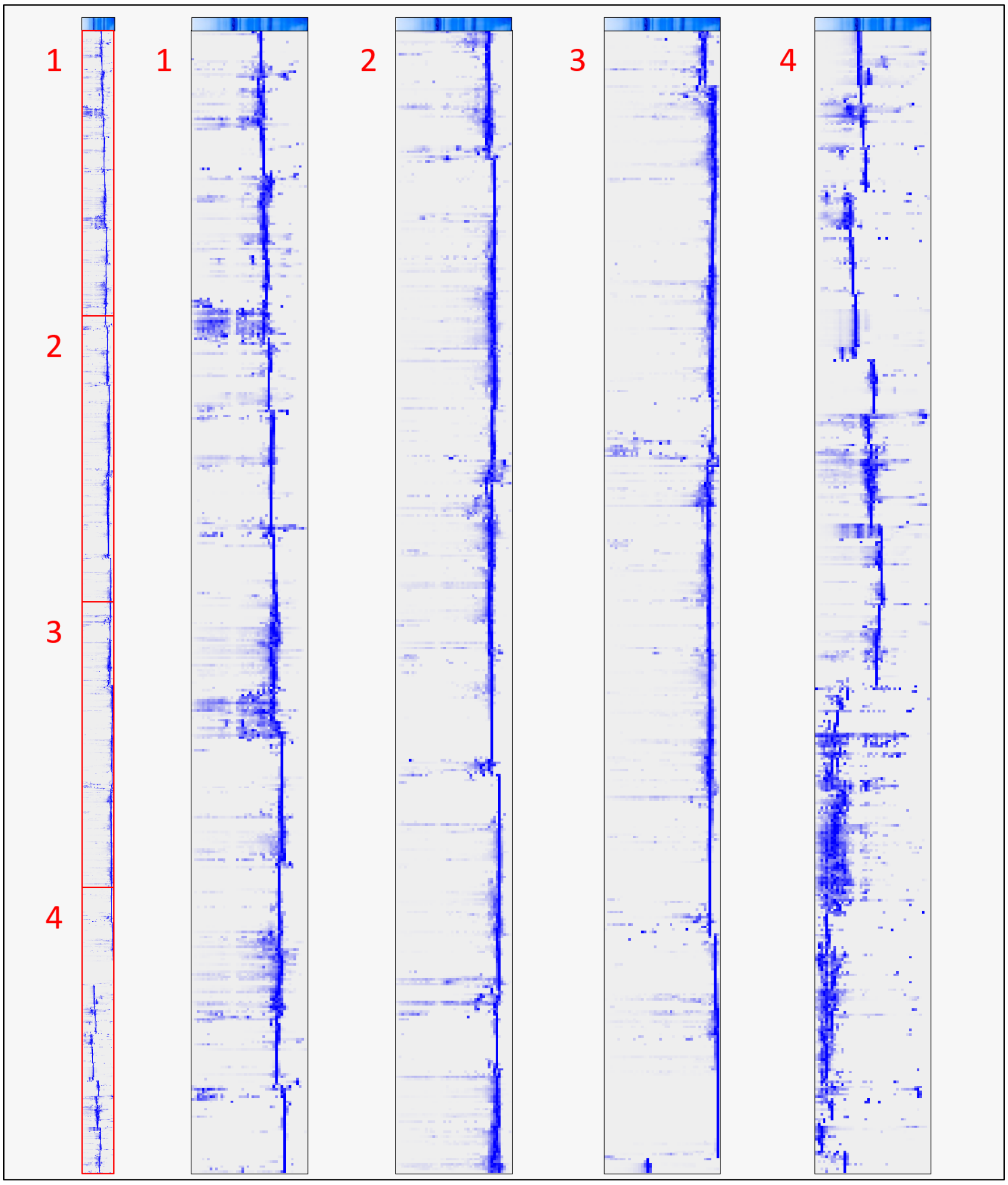

Supp. Fig. S6: Heat map of normalized (max) abundance profiles of thylakoid proteins from *V. album* leaves. The profiles were aligned by hierarchical clustering using the NOVA software (Giese et al. 2015). Left: entire heat map. The map consists of 44 columns corresponding to the 44 gel slices (left: fraction of largest molecular mass, right: fraction of lowest molecular mass) and 1,833 lines corresponding to the 1,833 identified unique proteins. Relative protein quantity is indicated by shades of blue (dark blue stands for high quantity, light blue/white for low quantity). Four figure parts to the right: enlarged sections of the entire map (the numbers correspond to the sections indicated in the entire map). The original BN gel is shown on top of all parts of the figure. For complete complexome profiling data see Supp. Data 1 (V. *album*) and Supp data 2 (*A. thaliana*). The map can be accessed in full detail at https://complexomemap.de/va_chloroplasts. A corresponding map has been generated for a *A. thaliana* thylakoid fraction and can be accessed at https://complexomemap.de/at_chloroplasts.
